# Real‐time assay of ribonucleotide reductase activity with a fluorescent RNA aptamer

**DOI:** 10.1002/1873-3468.70237

**Published:** 2025-12-01

**Authors:** Jacopo De Capitani, Noemi E. Nwosu, Viktoria Gocke, Müge Kasanmascheff, Hannes Mutschler

**Affiliations:** ^1^ Biomimetic Chemistry, Department of Chemistry and Chemical Biology TU Dortmund University Dortmund Germany; ^2^ Physical Chemistry, Department of Chemistry and Chemical Biology TU Dortmund University Dortmund Germany

**Keywords:** fluorescent light‐up aptamer, high‐throughput assay, nucleotide metabolism, ribonucleotide reductases

## Abstract

Ribonucleotide reductases (RNRs) convert all four ribonucleotides to deoxyribonucleotides, providing essential building blocks for DNA biosynthesis and repair through radical‐based catalysis. These functions are key to cellular proliferation and have made RNRs well established targets for antimicrobial and antiviral drugs and combination chemotherapies. Here, we describe a novel highly sensitive one‐pot enzymatic assay, which amplifies RNR activity by coupling it to the synthesis of a fluorogenic RNA aptamer. We validated this approach by testing RNR activity under dNTP‐limiting conditions to emulate RNR's complex allosteric regulatory patterns and by detecting the dose‐ and time‐dependent inhibition of RNR by hydroxyurea. This unique assay builds on previous high‐throughput screening assays for investigation of RNR's catalytic mechanisms by improving sensitivity and reducing readout timeframes.

**Impact statement**

Ribonucleotide reductases (RNRs) are essential for controlling cellular dNTP supply and are major targets in cancer, antiviral, and antimicrobial therapy. FLARE is a novel single‐tube, real‐time RNR assay, coupling dNTP synthesis to the transcription of a fluorogenic aptamer for continuous monitoring of activity, regulation, and inhibition using standard microplate readers.

## Graphical abstract

Ribonucleotide reductases (RNR) synthesize DNA building blocks *de novo*, making them crucial in DNA replication and drug targeting. FLARE introduces the first single‐tube real‐time coupled RNR assay, which enables isothermal tracking of RNR activity at nanomolar enzyme levels and allows the reconstruction of allosteric regulatory patterns and rapid screening of inhibitors.

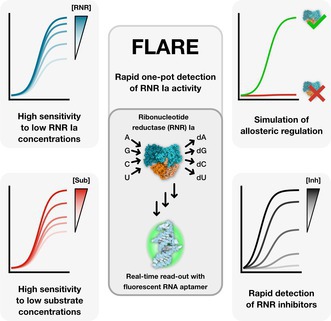

## Abbreviations

**CDP**, cytidine diphosphate

**dCDP**, deoxycytidine diphosphate

**dCTP**, deoxycytidine triphosphate

**dDTP**, mixture of dATP, dGTP, dTTP

**dNDP**, deoxynucleoside diphosphate

**dNTP**, deoxynucleoside triphosphate

**dsDNA**, double‐stranded DNA

**FLAP**, fluorescent light‐up aptamer

**FLARE**, Fluorescent Light‐up Aptamer RNR Enzymatic assay

**HTS**, high‐throughput screening

**HU**, hydroxyurea

**NDK**, nucleoside diphosphate kinase

**NDP**, nucleoside diphosphate

**NTP**, nucleoside triphosphate

**Phi29 DNAP**, Phi29 DNA polymerase

**RNR**, ribonucleotide reductase

**ssDNA**, single‐stranded DNA

**T7 RNAP**, T7 RNA polymerase

All living organisms depend on the availability of balanced deoxynucleotide (dNTPs) pools for DNA replication and DNA repair and ultimately for genome stability. Ribonucleotide reductases (RNRs) carry out the critical function of catalyzing the reduction of RNA building blocks to DNA building blocks in the only known biochemical pathway for *de novo* synthesis of dNTPs [1, 2]. For this reason, RNRs are a prerequisite for cellular proliferation and therefore RNRs are found in all free‐living organisms. [3]

Due to their conserved importance for DNA synthesis across organisms, RNRs have long been an interesting target for antimicrobial [4] and anticancer treatments. [5] Early structural studies [6, 7, 8, 9] first helped categorize diverse RNRs into three classes (I, II, III) and revealed a conserved mode of action across organisms. Understanding of RNR classes enabled the development of a first generation of therapies specifically inhibiting RNR. [10] However, more recent studies have resolved structures of trapped and inhibited RNR states at higher resolution [11, 12, 13] and thereby improved our understanding of RNR *in vivo* dynamics [14] and as targets for antibacterial, [15, 16, 17, 18] antiviral [19, 20] and anticancer [21, 22, 23, 24] treatments.

This ensemble of structural studies has revealed different oligomeric conformations across organisms but demonstrated a highly related topology between two subunits (α and β) in major RNR variants, observed, for instance, in the α_2_β_2_ heterodimer tetrameric conformation of class I RNRs. [1, 25] In their active state, RNRs catalyze the conversion of nucleoside 5′‐diphosphates (NDPs) or 5′‐triphosphates (NTPs) to deoxynucleotides (dNDPs or dNTPs) by a radical‐based catalytic mechanism that, in class I RNRs, involves a common active site architecture located in the α subunit and based on redox cycling of cysteines, [26] the formation of which requires the incorporation of metallo‐cofactors (e.g., Fe^2+^) in the β subunit and a long‐range radical transfer from the β to α subunits. [2, 27, 28, 29] While other RNR classes retain similar enzymatic functions, they employ distinct cofactors and assembly pathways [2, 25], such as the adenosylcobalamin cofactor [30] and the glycyl radical‐based mechanism, [31] for RNR class II and III, respectively. These conserved mechanisms allow RNRs to promiscuously catalyze the reduction of any NDP (or NTP) to their respective deoxy forms, by a sophisticated allosteric regulatory mechanism of substrate and effector binding to the α subunit, in which individual dNTPs function as the specificity effectors for the preferential reduction of specific nucleotides. [13, 32]

Improved understanding of the catalytic mechanism of RNRs has enabled the identification of new therapeutic targets. [33, 34] However, development of novel RNR inhibitors and advancements in understanding of RNRs have relied on methodologies that provide quantitative reliable measurements for RNR activity, but are either low‐throughput [35] or require large amounts of RNR for individual assay reactions. [36, 37] In contrast, assays that rely on NADPH absorption enable direct enzyme kinetics determination but have diminished sensitivity and require components that are not readily available. [13, 38] Furthermore, these assays have not been adopted to simulate the allosteric regulation of RNR Ia's catalytic activity as they have been primarily used for reduction of individual NDPs at a time and therefore have not shown reduction of multiple NDP substrates in a one‐pot assay. To address these constraints, high‐throughput screenings (HTS) of RNR activity based on mass spectrometry [39] or PCR‐based methods [15] have shown simultaneous reduction of multiple NDPs or have been used to screen multiple RNR inhibitors with increased sensitivity. However, these assays require labor‐intensive and time‐consuming multistep assaying procedures.

Here we present FLARE (Fluorescent Light‐up Aptamer RNR Enzymatic assay) a highly sensitive one‐pot rapid detection method of RNR multiturnover activity, which increases sensitivity and reduces processing times to investigate both allosteric regulation and inhibition of RNR. FLARE is designed to provide a rapid, microplate‐compatible and low‐enzyme set‐up, capable of high‐throughput profiling of allosteric regulation and inhibitory responses. Therefore, FLARE is intended to complement existing RNR enzymatic assays based on endpoint kinetic quench‐flow, mass spectrometry, or radioactive assays. To maximize sensitivity and signal‐to‐noise ratio achieved with the assay, we coupled the activity of the model enzyme RNR class Ia (i.e., RNR Ia) from *Escherichia coli* with transcription of the Broccoli RNA fluorescent light‐up aptamer (FLAP). [40] RNA FLAPs are short RNA molecules with a defined 3D structure that exhibit fluorescence upon binding a small molecule (e.g., DFHBI‐1T), by inducing, in the case of the Broccoli FLAP, the fluorescence of structural mimics of the HBI fluorophore found in GFP. [41] In this assay, the coupled reactions of the active RNR Ia α_2_β_2_ complex and nucleoside diphosphate kinase (NDK) produce dNTPs that are used by Phi29 DNA polymerase to generate the double‐stranded DNA (dsDNA) template, from which the Broccoli FLAP is transcribed. The coupled steps of dsDNA synthesis and RNA transcription, combined with Broccoli FLAP detection, drastically reduce assay times and increases sensitivity, allowing detection with low nanomolar RNR concentrations. Due to its design, involving downstream amplified readout of dNTP synthesis, FLARE is in principle agnostic to the RNR variant used. Therefore, FLARE is likely compatible with RNRs the *in vitro* activity requirements of which are known and can be used to adjust the composition of necessary cofactors and associated enzymes. Here, we show how we implemented FLARE to simulate *in vitro* the allosteric regulatory patterns of RNR Ia by detecting multiturnover reactions with multiple NDP substrates at once. To establish whether the assay could be used as a HTS method to screen potential RNR Ia inhibitors, we determined a dose–response inhibition of RNR Ia by hydroxyurea as proof‐of‐concept.

## Materials and methods

### DNA constructs

All DNA primers used for cloning were ordered from IDT (Integrated DNA Technologies, Coralville, IA, USA) and are listed in Table S1. The coding sequences of the proteins used in this study are listed in Table S2. The open reading frame of nucleoside diphosphate kinase (NDK) was isolated from an original expression vector (pQE30‐NDK‐His—Addgene ID:124136) with primer pair pr01 and pr02 and cloned into a pBAD33 backbone with the NEBuilder HiFi DNA Assembly Master Mix (New England Biolabs [NEB], Ipswich, MA, USA) to generate the resulting pBAD33‐NDK‐His expression vector for optimized protein expression. All cloning procedures were performed with chemically competent *E. coli* TOP10 cells. The identity of all final constructs was verified by sequencing (Microsynth AG, Balgach, Switzerland). The pET28b‐nrdA and pTB‐nrdB plasmids were kindly donated by JoAnne Stubbe and coworkers. The ssDNA Broccoli construct was designed with a T7 promoter immediately upstream of the core sequence of the Broccoli fluorescent light‐up aptamer, as described in more detail elsewhere. [40]

### Purification of NDK

Nucleoside diphosphate kinase was purified via affinity chromatography using an N‐terminal His6‐tag. The pBAD33‐NDK‐His construct was transformed into chemically competent *E. coli* TOP10 first on LB‐agar plates with 34 μg·mL^−1^ chloramphenicol (Cm) at 37 °C. Positive clones were then selected for subsequent overexpression and purification. The expression culture of Terrific Broth (TB) media, containing Cm, was induced at an OD600 = 0.6 with 0.2% w/v arabinose and grown overnight at 25 °C. Cells were harvested by centrifugation (6000 RCF for 30 min at 4 °C), resuspended in 40 mL per liter of culture in Buffer L (50 mm HEPES‐KOH pH 7.5, 250 mm NH_4_Cl, 10 mm MgCl_2_, 5 mm DTT, 20 mm imidazole, 1 mm PMSF) and lysed by sonication on ice. Cell debris was pelleted by centrifugation at 16500 RCF for 1 h at 4 °C. The clear supernatant was applied to 2 mL packed HisPur Ni‐NTA resin (Thermo Fisher Scientific, Waltham, MA, USA) per 1 L culture, equilibrated with buffer WA (50 mm HEPES‐KOH pH 7.5, 250 mm NH_4_Cl, 10 mm MgCl_2_, 5 mm DTT, 20 mm imidazole). After loading the supernatant to the resin, the resin was washed twice with five column volumes (CVs) buffer WA, twice with five CVs of buffer WB (same as buffer WA, but with 1 m NH_4_Cl) and again twice with five CVs of buffer WA. NDK was then eluted with three times three CVs of buffer E (same as buffer WA, but with 300 mm imidazole). Elution fractions were subsequently pooled and concentrated using an Amicon Ultra Centrifugal Filter Unit (Merck KGaA, Darmstadt, Germany) with a molecular weight cutoff of 10 kDa (centrifugation at 3260 RCF at 4 °C). After concentrating the elution fractions, the buffer was exchanged to buffer S (50 mm HEPES‐KOH pH 7.5, 100 mm KCl, 10 mm MgCl_2_, 10 mm TCEP, 30% Glycerol) by diluting the concentrate in the spin concentrator and then concentrating again, repeating this process 4 times and the concentration was estimated by UV–Vis with an extinction coefficient at 280 nm of 4470 m ^−1^ cm^−1^. The final protein preparation was then frozen in liquid nitrogen and stored at −80 °C.

### Expression and purification of *E. coli* RNR Ia α2

Purification of RNR Ia α2 dimer (NrdA) was performed as previously described. [42, 43] If not stated otherwise, 50 mm Tris‐HCl buffer containing 5% glycerol and 2 mm DTT at pH 7.6 was used for all purification steps, shortly termed *TrisA*. *E. coli* BL21(DE3)‐Gold were transformed with pET28a‐nrdA and plated on LB‐agar plates with 50 μg·mL^−1^ kanamycin (Km) at 37 °C. The expression culture of LB medium containing Km was induced with 0.5 mm IPTG at OD600 = 0.6 and harvested by centrifugation at 17000 RCF for 15 min at 4 °C after 5 h of overexpression. The cell pellet was resuspended in 5 mL·g^−1^ cell pellet with *TrisA* buffer, supplemented with 1 mm PMSF and lysed via four passages through an EmulsiFlex‐C5 cell homogenizer (Avestin, Inc., Ottawa, ON, Canada). Insoluble cell debris was removed at 3200 RCF for 30 min at 4 °C. DNA was precipitated by adding 0.2 vol eq. of a 6% (w/v) streptomycin solution dropwise, followed by centrifugation at 3200 RCF for 30 min at 4 °C. The clear supernatant was loaded onto a pre‐equilibrated Ni‐Sepharose High Performance column (Cytiva, Marlborough, MA, USA). *TrisA* buffer containing 20 mm imidazole and 200 mm NaCl was used for equilibration. The column was first washed with the same buffer, and the protein was eluted by raising the imidazole concentration to 250 mm. The eluted protein was precipitated by 60% saturation with (NH_4_)_2_SO_4_ (39 g/100 mL). The salt was slowly added for over 10 min and stirred for an additional 15 min on ice. Then, the protein was isolated at 3200 RCF for 30 min at 4 °C and resolubilized in a minimal amount of *TrisA* buffer. Desalting was performed using a PD‐10 column (Sephadex G‐25 Medium, Cytiva), pre‐equilibrated with *TrisA* buffer. The purified protein was concentrated in an Amicon centrifugal filter unit with 30 kDa cutoff (Merck KGaA), and the concentration was estimated via UV–Vis with an extinction coefficient at 280 nm of 189 000 m ^−1^ cm^−1^. Aliquots were flash frozen in liquid nitrogen and stored at −80 °C. A typical yield of 2–4 mg protein·g^−1^ cell paste was obtained.

### Expression and purification of *E. coli* RNR Ia apo‐β2

Purification of RNR Ia apo‐β2 dimer (NrdB) was performed as previously described. [42, 43] If not stated otherwise, 50 mm Tris‐HCl buffer containing 5% glycerol at pH 7.6 was used for all purification steps, shortly termed *TrisB*. *E. coli* BL21(DE3)‐Gold were transformed with pTB‐nrdB and plated on LB‐agar plates with 100 μg·mL^−1^ carbenicillin (Carb) at 37 °C. The expression culture of LB medium containing Carb was treated with 100 μm 1,10‐phenanthroline to chelate iron at OD600 = 0.9 and induced with 0.5 mm IPTG after 20 min. After 20 h of protein overexpression, the cells were harvested by centrifugation at 17 000 RCF for 15 min at 4 °C. The cell pellet was resuspended in 5 mL·g^−1^ cell pellet with *TrisB* buffer containing 0.5 mm PMSF. The suspension was lysed via four passages through an Emulsiflex‐C5 cell homogenizer (Avestin, Inc.). Insoluble cell debris was removed at 3200 RCF for 30 min at 4 °C. DNA was precipitated by adding 0.2 vol eq. of a 6% (w/v) streptomycin solution dropwise, followed by centrifugation at 3200 RCF for 30 min at 4 °C. NrdB protein was precipitated by 60% saturation with (NH_4_)_2_SO_4_ (39 g/100 mL) by slowly adding the salt for over 10 min and stir for an additional 15 min on ice. The protein was isolated at 3200 RCF for 30 min at 4 °C and resolubilized in a minimal amount of *TrisB* buffer. Desalting was performed using a PD‐10 column (Sephadex G‐25 Medium, Cytiva), preequilibrated with *TrisB* buffer. Protein‐containing fractions were then loaded onto a pre‐equilibrated anion exchange column (DEAE Sepharose Fast Flow, Cytiva), connected to a cooled chromatography system. Equilibration and subsequent washing of the column were performed with *TrisB* buffer, enriched with 100 mm NaCl. Protein elution was carried out with a gradient of 100 to 500 mm NaCl. Protein‐containing fractions were pooled and diluted with an equal volume of *TrisB* buffer to avoid high salt concentrations and purified via a second anion exchange column (Q Sepharose Fast Flow, Cytiva). *TrisB* buffer supplemented with 150 mm NaCl was used for equilibration and washing of the column, and elution was performed with a NaCl gradient from 150 to 500 mm. Protein concentration was checked via UV–Vis, and EDTA was added to the solution to a final concentration of 100 times the protein concentration, followed by incubation for 2 h at 4 °C. EDTA was removed by washing with buffer *TrisB* in an Amicon centrifugal filter unit with 30 kDa cutoff (Merck KGaA) several times. After EDTA removal and reaching the desired protein concentration, aliquots were frozen in liquid nitrogen and stored at −80 °C. A typical yield of 9–18 mg protein·g^−1^ cell paste was obtained. For the apoprotein, an extinction coefficient at 280 nm of 120 000 m ^−1^ cm^−1^ was used for concentration estimation.

### Generation of RNR Ia β2 Tyr radical

Before any use, to ensure incorporation of Fe(II) and generation of the Tyr radical, 30 μm of purified RNR apo‐β2 was treated with 5 equivalents of ammonium iron(II) sulfate (prepared fresh each time) and left to incubate on ice in an open reaction tube for 10 min to ensure sufficient oxygenation. Formation of the Tyr radical (Y122·) was confirmed in either 50 mm Tris/HCl pH 7.5 or 1× RNR reaction buffer by measuring the UV–vis absorbance across the 350–500 nm range. Y122· radical yield was quantified by the dropline correction method based on the characteristic absorption maximum at 410 nm (Eq. 1). [44, 45]

(1) $$C_{Y_{122}}=\frac{A_{410}-0.5\left(A_{400}+A_{415}\right)}{2110\;M^{-1}{\mathrm{cm}}^{-1}}\cdot \text{dilution}$$

### PCR‐based assay of RNR Ia activity

Reactions with a PCR‐based readout of RNR Ia activity were carried out with the PCR‐based assay by Tholander et al. [15] Before assembling the assay reactions, purified RNR apo‐β2 was treated with ammonium iron(II) sulfate (prepared fresh each time) to ensure generation of Tyr radical. [46] Assay reactions with RNR Ia purified from *E. coli* were prepared in a final reaction volume of 30 μL. About 1 μm NrdA and 1 μm NrdB were mixed with 30 mm Tris‐HCl pH 7.5, 20 mm Mg(OAc)_2_, 1.5 mm CHAPS, 1.5 mm DTT, 5 mm ATP, and 1% DMSO. To identify the optimal concentration of reducing agent, varying concentrations of TCEP (0–5 mm) were added to separate reactions. After a 30 min incubation at 37 °C, 200 μm CDP were added to the reactions. Samples were then incubated at 25 °C for 2 h and then quenched by heating at 70 °C for 10 min. After letting the samples cool to room temperature, 115 nm NDK were added to the reactions. Reactions were then incubated at 25 °C overnight and then quenched by heating at 70 °C for 10 min. The RNR reaction mixes were combined with dCTP‐deficient PCR mixes (1× Q5 HF Buffer (NEB), 200 μm of each dATP, dGTP and TTP, 500 nm of each primer pr06 and pr07, 1 ng pUC19 plasmid, and 0.02 U·μL^−1^ Q5 HotStart HF DNA Polymerase (NEB)) (30% RNR reaction mix, 70% PCR mix, in a final volume of 25 μL). The PCR products (137 bp) were visualized on 3% TAE agarose gels, and band volumes were quantified using ImageLab (Bio‐Rad) and normalized with min–max scaling. The normalized band volumes were then fit to a bi‐phasic dose response model to estimate optimal TCEP concentration (Eq. 2), where *y*_0_ is the initial *y* value, *y*_max_ is the maximum *y* value, *y*_end_ is the final *y* value, TA50 is *x* value at 50% of the activation curve, TI50 is the *y* value at 50% of the inactivation curve, Ha is the activation Hill coefficient (slope of activation), and Hi is the inactivation Hill coefficient (slope of inactivation).

(2) $$Y=y_{0}+\frac{y_{\max}-y_{0}}{\left(1+10^{\left(\mathrm{TA}50-x\right)*\mathrm{Ha}}\right)}+\frac{y_{\mathrm{end}}-y_{\max}}{\left(1+10^{\left(\mathrm{TI}50-x\right)*Hi}\right)}$$

### Real‐time RNR activity assay with broccoli RNA aptamer – FLARE

Before assembling the assay reactions, purified *E. coli* class Ia RNR apo‐β_2_ was treated with 5 equivalents of ammonium iron(II) sulfate (prepared fresh each time) to ensure tyrosyl radical (Y_122_·) formation. Assay reactions with RNR Ia purified from *E. coli* were prepared in a final reaction volume of 15 μL. Each reaction contained 1× of RNR reaction buffer (50 mm Tris/HCl pH 7.5, 30 mm Mg(OAc)_2_, 1.5 mm DTT, 10 mm NaCl, 2 mm spermidine, assembled in a 10× reaction buffer—Table S3) which was designed based on previously described buffer requirements of RNR Ia [15] and manufacturer's specifications for the other protein components. Additional components included in the reactions were rNTP mix (Table S4, final concentrations in reaction of 5 mm ATP, 2 mm GTP, CTP, UTP—Jena Bioscience GmbH, Jena, Germany), 1.5 mm TCEP (tris(2‐carboxyethyl)phosphine) (Biotium, Fremont, CA, USA) and 10 μm DFHBI‐1T [(Z)‐4‐(3,5‐difluoro‐4‐hydroxybenzylidene)‐2‐methyl‐1‐(2,2,2‐trifluoroethyl)‐1H‐imidazole‐5(4H)‐one] (Biomol GmbH, Hamburg, Germany). 10 μm DFHBI‐1T was chosen as increasing the concentration did not lead to a significant increase in fluorescence intensity (Fig. S1). Each reaction also contained the following protein components: 1 U·μL^−1^ Murine Rnase Inhibitor (NEB), 115 nm *E. coli* nucleoside diphosphate kinase (NDK), 200 nm T7 RNA polymerase (Thermo Fisher Scientific), 1 μm purified RNR Ia α_2_, 1 μm purified RNR Ia β_2_ Ia and 10 U Phi29 DNA polymerase (NEB). Finally, for the readout, each reaction contained 500 nm of Broccoli ssDNA and 500 nm primer pr05.

This general reaction framework was then used as the basis to test different combinations of nucleoside diphosphates (NDPs) (CDP—TCI Chemicals, Tokyo, Japan, ADP—Jena Bioscience, GDP and UDP—Sigma‐Aldrich, St. Louis, MO, USA) and deoxynucleoside triphosphates (dNTPs) (Thermo Fisher Scientific). NDPs were prepared fresh each time before use and 2 mm for each NDP were supplied in different combinations to the reaction; 200 μm of the dNTP version of each nucleobase not supplied as NDP were supplied to the reaction (e.g., if 2 mm CDP was supplied to the reaction, 200 μm of each dATP, dGTP, and dTTP were supplied on top). Reactions were gently mixed by pipetting and then incubated at 30 °C in a StepOne Real‐Time PCR System (Thermo Fisher Scientific) for 4 to 6 h depending on experimental requirements. Fluorescence measurements (excitation: 488 nm, emission 510–530 nm) were taken every 6 min to reduce photobleaching of the DFHBI‐1 T dye. Reaction components for an example reaction are summarized in Table S5.

The raw data were normalized with min–max scaling between the averages of the internal negative and positive controls (i.e., ∆RNR, dDTPs—negative; ∆RNR, dNTPs—positive). Normalized data were then used to determine the apparent rate.

### Determination of apparent reaction rate in real‐time assay

The raw RFU data from FLARE reactions were normalized with min–max scaling between the averages of the internal negative and positive controls (i.e. ∆RNR, dDTPs—negative; ∆RNR, dNTPs—positive). The normalized RFU data were then smoothened with a Savitzky–Golay filter to reduce noise in the data without distorting data tendency. The filtered data were then fit to the scaled logistic function $Y=\frac{L}{1+e^{-k\left(x-x_{0}\right)}}+b$ where *L* is the curve's asymptote, *k* is the steepness of the curve, *x*_0_ is the midpoint of the logistic function, and *b* is the *y*‐offset of the curve. The second derivative of the fitted logistic function was computed to estimate the coordinates of the critical points. The local maximum of the derivative was defined as the end of the lag phase in the reaction signal, while the local minimum was the end of the log phase of exponential fluorescence increase. A linear regression model was then fit to the smoothened data between the *x* coordinates of the second derivative's critical points. The apparent rate of the reaction is then defined as the slope of the fitted linear model. Lower conversion rates of NDP substrates into dNTPs result in lower transcription rates of the Broccoli RNA aptamer and therefore lower apparent rates in the log phase. This method was preferred to numerical determination of local maxima and minima results by determining central differences of the smoothened data over time due to unclear determination of the beginning and end of log phase of reaction. An example of apparent rate determination is shown in Fig. S2. The python code used to calculate the apparent FLARE reaction rates can be found in File S1. The source data for the data presented in this study can be found in File S2.

### Hydroxyurea dose–response analysis with FLARE

Dose–response of hydroxyurea (HU) was performed by pre‐incubating RNR Ia with increasing concentrations of HU. RNR Ia apo‐β_2_ was loaded with 5 equivalents of ammonium iron(II) sulfate. 10 μm of each RNR Ia subunit was incubated with HU following a linear 10‐fold titration (0–100 mm) for 30 min at 37 °C. 2 mm CDP was then added, and reactions were incubated for 2 h at 25 °C, followed by a 10 min heat‐inactivation step at 70 °C. The RNR Ia ‐ HU mixture was then added to the dCTP‐limiting FLARE assay mixture for a final concentration of RNR Ia of 1 μm. To test in real‐time the time‐dependent inhibitory effect of HU on RNR Ia, 1 μm of each RNR Ia subunit (β2 was preloaded with 5 equivalents of Fe^2+^) and 3 mm of HU were added simultaneously to dCTP‐limiting FLARE reactions.

The assembled FLARE assay reactions were incubated at 30 °C for 6 h in a StepOne Real‐Time PCR System (Thermo Fisher Scientific), while measuring fluorescence as described above. For the pre‐incubation sample set, the apparent rates at different HU concentrations were then fit to a 4‐parameter logistic dose–response model $\frac{E_{\max}+\left(E_{\max}-E_{\min}\right)}{1+{\left(\frac{\mathrm{IC}50}{x}\right)}^{b}}$ where *E*_max_ is the upper plateau, *E*_min_ is the lower plateau, IC50 is the HU concentration at 50% response, and *b* is the slope.

## Results

### Optimizing readout of RNR Ia's activity with Broccoli FLAP

**Fig. 1:**
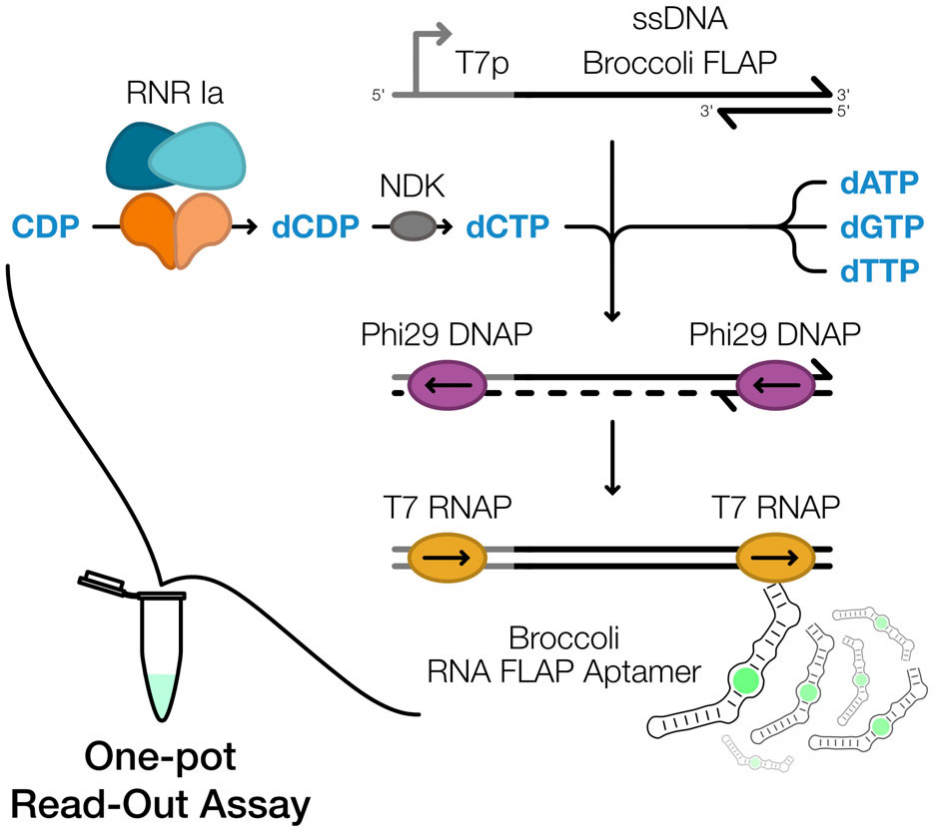
Schematic illustration of one‐pot real‐time detection of RNR Ia activity with Fluorescent Light‐up Aptamer RNR Enzymatic assay (FLARE). Starting from a partially double‐stranded DNA template that encodes for the Broccoli RNA fluorescent light‐up aptamer (FLAP), the coupled consecutive reactions of ribonucleotide reductase (RNR) Ia (blue and orange), nucleoside diphosphate kinase (NDK) (gray), Phi29 DNA polymerase (DNAP) (purple) and T7 RNA polymerase (RNAP) (yellow) allow readout by quantification of fluorescence emitted by the Broccoli FLAP – DFHBI‐1T complex. The schematic shows the reduction of cytidine diphosphate (CDP), coupled with the supplementation with dDTPs (i.e., the mixture of dATP, dGTP, dTTP), as an example of the functionality of the assay.

To establish a proof of concept of the FLARE assay's functionalities, we coupled the RNR class Ia ‐dependent reduction of cytidine 5′‐diphosphate (CDP) with the transcription of the Broccoli RNA FLAP (from here referred to as Broccoli). RNR Ia reduces CDP to dCDP (deoxycytidine 5′‐diphosphate), which is then phosphorylated by NDK to dCTP (deoxycytidine 5′‐triphosphate). This coupled reaction generates the substrate for Phi29 DNA polymerase (DNAP), which, together with the other dDTPs (i.e., the mixture of dATP, dGTP, dTTP), fills‐in a partially double‐stranded (ds) DNA template encoding for the Broccoli aptamer template. Completion of this template establishes a functional T7 promoter (T7p), enabling multiturnover T7 RNA polymerase (RNAP)‐mediated transcription of the Broccoli aptamer (Fig. 1). The transcribed Broccoli binds the fluorogen DFHBI‐1T, resulting in detectable green fluorescence upon excitation with blue light. Thus, the intended goal of the FLARE assay is the development of a simple yet sensitive readout system for RNRs that enables comparative evaluation of dNTP production.

To establish a new readout method for RNR Ia activity, assay conditions first had to be optimized to accommodate the specific requirements of RNR Ia and of the additional protein components. First, the buffer system for the assay was designed taking into consideration each protein's salt, pH, and cofactor requirements, and assuring that the Y_122_· radical in the β subunit could be generated in the designed buffer system. [44, 45] Prior to assays, apo‐β_2_ was reconstituted with Fe^2+^ and Y_122_· formation was verified by UV–vis (410 nm) and quantified by dropline correction at 410 nm, yielding 61% loading in Tris‐HCl and 49% in the 1× RNR reaction buffer (Fig. S3). The observed values of Fe^2+^ loading in β_2_ are consistent with previously reported values, which show that β_2_ activity is limited to at most 60% active β_2_ and with at most 3.6 eq of iron atoms bound, regardless of Fe^2+^ availability. [44, 45] Second, RNR Ia multiturnover catalysis relies on the effective reduction of conserved cysteines in the active site in the α subunit after each round of NDP reduction; while *in vivo* this is achieved by thioredoxin and glutaredoxin [2, 26], similar effects can be recreated *in vitro* with a reducing agent (e.g., DTT or TCEP). [47] While DTT can act as a reductant on the cysteines in the active site itself, TCEP more closely resembles the biological mechanism of thioredoxin by reducing the C‐terminal cysteines of the α subunit, which then generate the active site. [47] Therefore, we tested the concentration‐dependency of TCEP, alongside 1.5 mm DTT, of RNR Ia activity with a dCTP‐limiting PCR‐based readout. [15] With this assay, we observed a bell‐shape response, with higher RNR activity detected at 1.5 mm TCEP (Fig. S4). [15, 47] This screening also underscored practical constraints associated with existing RNR HTS enzymatic assays: although they allow the simultaneous testing of multiple reaction conditions, they typically involve several steps across multiple days. Our intention was therefore to devise a highly sensitive RNR HTS assay with faster sample processing times and the ability to measure RNR Ia's activity within hours.

### FLARE detects RNR Ia activity in dCTP‐limiting set‐up

**Fig. 2:**
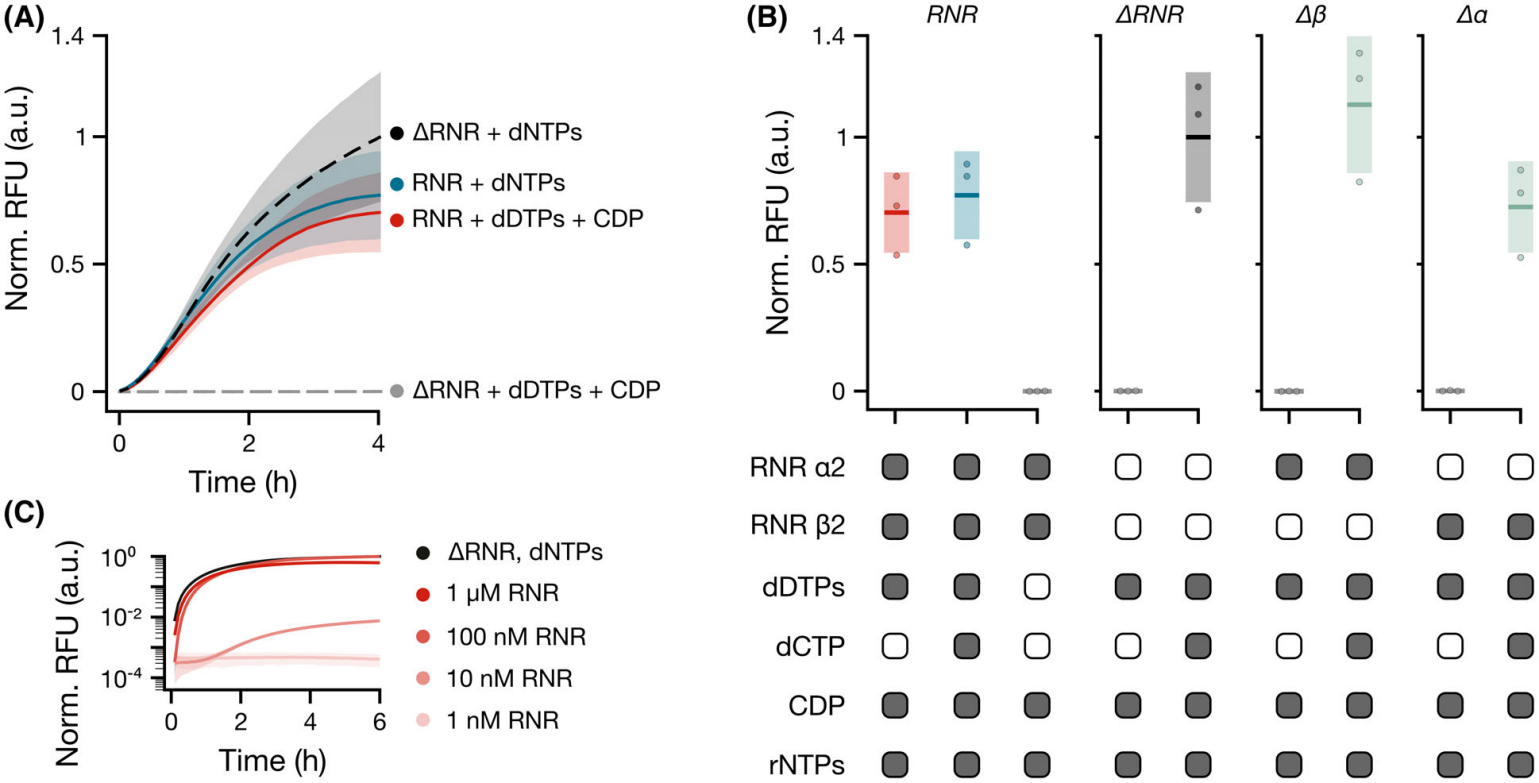
Proof‐of‐concept of FLARE assay. (A) Detection of RNR Ia‐dependent fluorescence emission (i.e., normalized relative fluorescence units – Norm. RFU) from Broccoli RNA fluorescent light‐up aptamer (FLAP) measured over time. FLARE reactions were carried out in the absence (dashed lines) or presence (solid lines) of 1 μm of ribonucleotide reductase (RNR) Ia subunits and supplemented with either 200 μm dNTPs or 2 mm cytidine diphosphate (CDP) and 200 μm dDTPs (i.e., mixture of dATP, dGTP, and dTTP). (B) Normalized maximum RFU values from different combinations of assay components and RNR Ia subunits. In the presence of both RNR Ia subunits, dCTP (deoxycytidine triphosphate) is produced upon reduction of CDP to dCDP (deoxycytidine diphosphate), which is then rephosphorylated by nucleoside diphosphate kinase (NDK) to dCTP. FLARE signals are observed only when functional RNR Ia, CDP and dDTPs are present, or when all dNTPs are supplied to the reaction. The full set of experimental controls can be found in Fig. S5. In the sample matrix, components present in the reactions are marked in dark gray and absent components in white. (C) Detection over time of RFU values with titration of RNR Ia concentrations (1 μm–1 nm). 2 mm CDP were supplied to the reactions alongside 200 μm dDTPs (apart from positive control—black—which is supplemented with 200 μm dNTPs). For all panels: independent replicates; *n* = 3, mean and SD shown. Reaction measurements were normalized with min–max scaling between the averages of the maximum fluorescence readouts of ∆RNR‐dNTPs samples and t0 measurement of ∆RNR‐dDTPs‐CDP samples.

As anticipated, initial experiments demonstrated that RNR Ia and NDK could produce sufficient dNTPs for Phi29 DNAP to generate the full‐length dsDNA template for Broccoli transcription by T7 RNAP. In the absence of exogenous dCTP, RNR Ia and NDK were capable of rapidly generating the necessary deoxynucleoside triphosphate from CDP in amounts sufficient for Phi29 DNAP to produce the Broccoli dsDNA template (Fig. 2A). In contrast, no transcription of Broccoli was observed, when both or either of each RNR subunit were omitted from the experiment (Fig. 2A,B, Fig. S5).

The coupled conversion from CDP to dCTP was not rate limiting for Broccoli transcription, as no significant difference was observed in the fluorescence time traces of samples supplied with either all four dNTPs or dDTPs only (i.e., CDP with dATP, dGTP, and dTTP). Notably, the amplification of RNR Ia activity by transcription of the FLAP increased the assay sensitivity for low nanomolar concentrations of RNR Ia, enabling a qualitative activity assessment down to 10 nm of active RNR Ia, albeit while partially compromising on readout speed (Fig. 2C, Fig. S6). After confirming that robust fluorescence signals were observed with 2 mm CDP and 200 μm dDTPs, we used a dCTP‐limiting set‐up to determine the reliable detection range of the assay and to assess FLARE's sensitivity depending on substrate concentrations. We therefore challenged FLARE by varying the concentrations of either CDP or dDTPs. When titrating CDP concentrations with a constant 200 μm dDTPs input, we detected FLARE readouts, comparable to the standard 2 mm input, down to 3.2 μm CDP (Fig. S7). When limiting the dDTPs concentrations at a constant 2 mm CDP input, we observed an unambiguous fluorescence readout down to 0.32 μm dDTPs. Under these conditions, both maximum observed fluorescence and apparent reaction rates plateaued at 8 μm and remained constant up to 200 μm dDTPs. The FLARE assay therefore reliably amplified the activity of RNR Ia with extreme sensitivity to both low‐enzyme and substrate concentrations without any detectable background signal due to its high specificity.

As the timepoints at which fluorescence peaked varied depending on sample composition, we identified the apparent rate of the exponential phase of the reactions as a reliabe method to compare RNR‐dependent readout in the FLARE reactions (Fig. S2). Although there was generally a good correlation (Fig. S5) between the maximal fluorescence readouts and the apparent rate (e.g., due to higher RNR concentrations; see Fig. S6), the determined apparent rates allowed for a robust comparison between different samples that exhibited maximum fluorescence at different timepoints. Since FLARE relies on a coupled, amplified readout, the apparent fluorescence rates extracted from the exponential phase report cumulative multiturnover activity and track the relative activity of the coupled reactions required to elicit a fluorescent signal and therefore are not direct *k*_cat_ values of RNR Ia per active cofactor.

### FLARE enables readout of allosteric regulation of RNR Ia

**Fig. 3:**
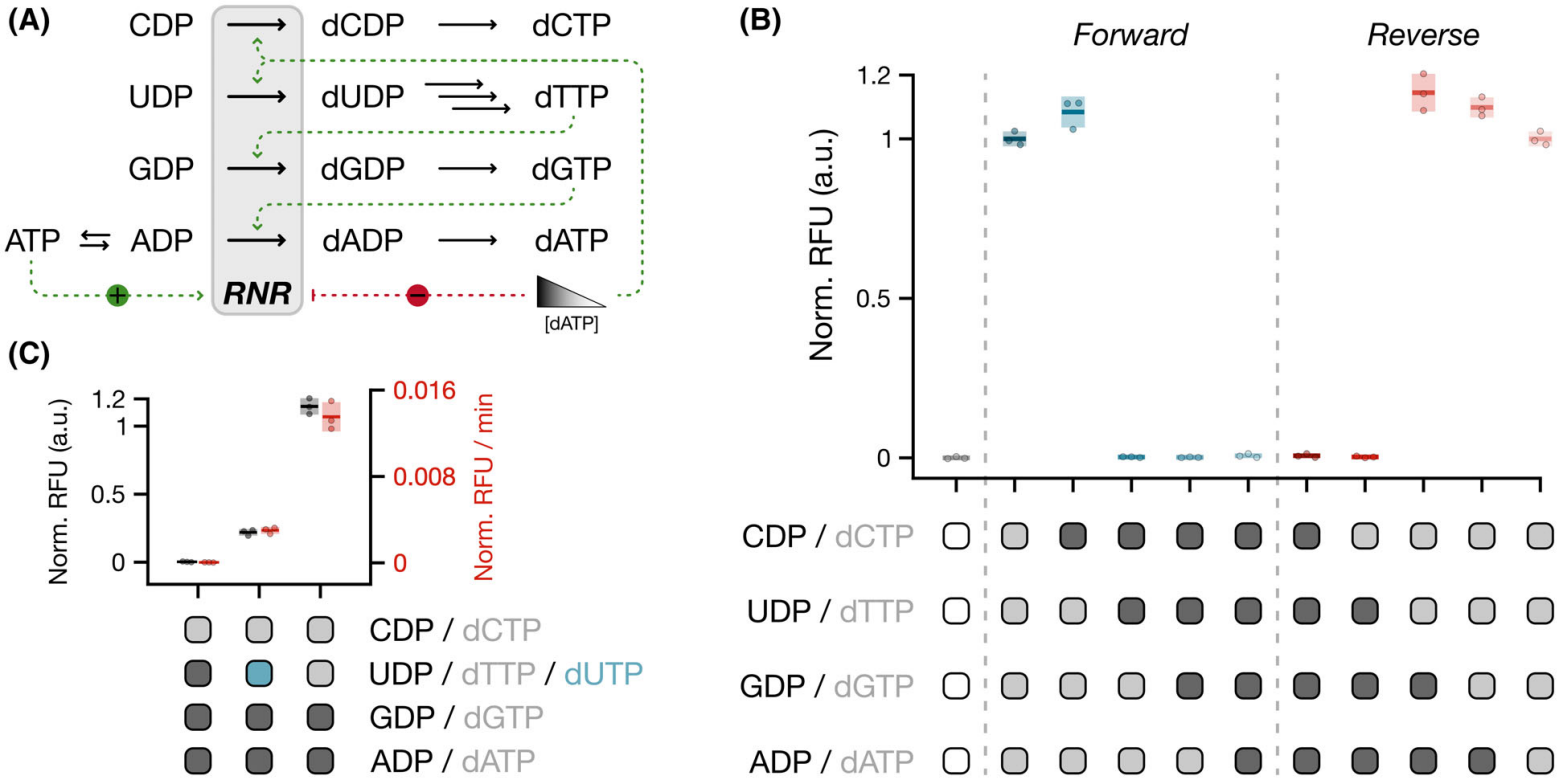
Investigating RNR Ia allosteric regulation of NDP reduction patterns with FLARE. (A) Schematic of ribonucleotide reductase (RNR) Ia reduction cascade based on differential allosteric regulation by previously reduced nucleotides. Allosteric regulations that activate the reduction of specific nucleoside diphosphates (NDPs) are highlighted in green, while regulations that inhibit RNR Ia activity are highlighted in red. Higher concentrations of dATP (deoxyadenosine triphosphate) inhibit RNR Ia activity, while lower concentrations act as specificity effector for the reduction of CDP and UDP (gray triangle). (B) Normalized maximum relative fluorescence units (Norm. RFU) values for either forward or reverse cascades. Depending on the reaction composition, to generate a fluorescent readout, 2 mm of each of the necessary NDPs (dark gray) are supplied to reactions alongside 200 μm of each of the complementing dNTPs (light gray). 1 μm of each RNR Ia subunit was included in the reactions. (C) Maximum normalized RFU values and apparent rates of the dCTP‐limiting assay, to determine FLARE readout based on the supplied UDP version. In each reaction, 2 mm CDP, 200 μm dATP, and 200 μm dGTP were supplied alongside either 2 mm UDP, 200 μm dUTP, or 200 μm dTTP. For measurements in panels B and C: technical replicates; *n* = 3, mean and SD shown. Reaction measurements were normalized with min–max scaling between the averages of the maximum fluorescence readouts of ∆RNR‐dNTPs samples and t0 measurement of ∆RNR‐∆dNTPs samples.

After establishing that RNR Ia could provide a model substrate (i.e., dCTP) for detection via Broccoli readout, we set out to investigate the tolerance of RNR Ia for different combinations of NDP substrates. To this end, we emulated how depletion of dNTPs during DNA synthesis may lead to an excess of the respective NDPs, which then need to be reduced. To do this, we recreated *in vitro* the allosterically regulated reduction pattern by RNR Ia, described by *in vitro* mechanistic studies. [1, 13] According to this ‘reduction cascade’ (Fig. 3A), each NDP, after being reduced to its deoxy form and subsequently phosphorylated to its triphosphate form, acts as the specificity effector for the reduction of the next nucleotide in the cascade, depending on relative concentrations of each dNTP. [13] ATP acts as the activity effector, which ensures that RNR Ia maintains its active α_2_β_2_ conformation. [38, 48] Under intracellular conditions, low concentrations of dATP promote the reduction of CDP and UDP. The conversion of dUDP to dTTP ensures that RNR can then reduce GDP, and the generation of dGTP triggers the conversion of ADP to dATP. Gradually increasing concentrations of dATP displace ATP from the activity site and inhibit RNR Ia's activity.

Before recreating the allosteric regulatory patterns of RNR Ia *in vitro* with the FLARE assay, we first determined the reliability of the assay to detect reduction of all individual NDP substrates. When the assay was tested with limiting amounts of each individual dNTP (Fig. S8A), Broccoli was generated at similar rates. Hence, all individual NDPs could be reduced with similar efficiencies in the respective dNTP‐limiting set‐ups. For example, when CDP alone had to be reduced, we supplied dDTPs; alternatively, when ADP alone had to be reduced, we supplied dBTPs (i.e., the mixture of dGTP, dTTP, dCTP). This demonstrated that the assay is sensitive enough to generate a comparable signal for each nucleotide despite differences in content of each base in the ssDNA template (Fig. S8B). Furthermore, since the generation of dUTP produced a similar signal to other dNTPs, Phi29 DNAP proved to be able to tolerate higher concentrations of dUTP as a substrate alternative to dTTP for this assay (Fig. S9).

FLARE reactions meant to emulate the allosteric regulation of RNR Ia in the presence of multiple NDPs were initiated with different combinations of NDPs and dNTPs to simulate the reduction cascade, where each combination represented an individual step in the cascade. By following the allosteric regulatory pattern of RNR Ia with either ‘forward’ or ‘reverse’ cascades, we emulated how replacing dNTPs with NDPs changed the metabolic burden on RNR Ia and therefore changed the conditions necessary to generate a positive readout signal. The *forward* cascade was designed to directly resemble the *in vivo* sequential substitution of dNTPs by NDPs. In this set‐up, each nucleotide was first supplied in its dNTP form, followed by a substitution of dCTP by CDP, then dTTP by UDP, and so on until only NDPs were supplied directly to the reaction. With this set‐up, we could recreate the established role of dTTP as allosteric regulator for GDP, which cannot be substituted with dUTP. In reactions where CDP alone had to be reduced, Broccoli was readily synthesized. However, as soon as dTTP was removed from the mixture, no signal could be observed (Fig. 3B, Fig. S10). [13] This behavior was confirmed in the *reverse* cascade set‐up. In this case, each nucleotide was supplied in its NDP form and then substituted by its dNTP version (i.e., CDP to dCTP, UDP to dTTP, and so on). Also in this case, signals were observed only in reactions with dTTP instead of UDP (Fig. 3B, Fig. S10).

We found that the increased sensitivity of the FLARE assay enabled us to detect the reduction of multiple NDPs by RNR Ia simultaneously, despite the enzyme's complex allosteric regulation, increased catalytic burden, and rate‐limiting dsDNA synthesis resulting from the lower supply of dNTPs. For instance, when VDPs (i.e., the mixture of ADP, GDP, and CDP) and dTTP were introduced in a dVTP‐limiting assay, the detected fluorescent signal was comparable to the reference dTTP‐limiting assay (i.e., assay supplied with UDP and dVTPs—dATP, dGTP, dCTP) (Fig. S11A), albeit with marginally slower apparent rates (Fig. S11B). This can be again explained by the fact that FLARE does not account for the conversion of dUTP to dTTP carried out by dUTPase and thymidylate synthase *in vivo*. [49] The reduced affinity for dUTP by RNR Ia as the specificity effector for reduction of GDP compared to dTTP, [11, 13, 32] likely hinders reduction of purines, when GDP and ADP reduction is dependent on prior dUTP conversion to dTTP. This would therefore contribute to lower concentrations of dsDNA template for Broccoli transcription and ultimately lower readout signals and slower observed apparent rates. In a dRTP‐limiting assay (i.e., requiring the synthesis of dATP and dGTP) with dUTP supplied to the reaction instead of dTTP, this effect was partially offset by introducing higher concentrations of dUTP compared to what the system could produce by itself when reducing multiple NDPs (Fig. 3C). This suggests that RNR Ia could use dUTP as specificity effector for GDP reduction, albeit with lower affinity. The combination of these results reflected the complex allosteric regulation of RNR Ia when using UDP as a substrate in combination with other NDPs and helped confirm the enzyme's regulatory mechanisms in a complex protein reaction network with sequential reduction of multiple NDPs. The observed behavior indicates that dUTPase and thymidylate synthase would be required to complete the dTTP metabolic pathway due to RNR Ia's intricate allosteric control.

### Detecting RNR Ia inhibition by hydroxyurea with FLARE

After confirming the versatility of the FLARE assay with a variety of NDP substrates, we focused on evaluating its reliability as an HTS tool for RNR Ia inhibitory compounds. As proof‐of‐concept, we screened RNR Ia activity upon treatment with hydroxyurea (HU). HU is an established cost‐effective treatment targeting RNR Ia in several diseases. [50, 51] With its dose‐ and time‐dependent mechanism, [52] HU acts as a suicide inhibitor that prevents NDP reduction by reducing the Y_122_· radical in the β subunit, [53, 54] by intercepting a pathway radical at the α/β subunit interface, [55] and its effect is potentiated in the presence of on the active α_2_β_2_ complex and its cofactors (i.e., Fe^2+^ for RNR Ia). [56]

We first tested the dose‐dependent inhibitory effect of HU on RNR Ia by pre‐incubating the α_2_β_2_ complex, loaded with Fe^2+^, for 30 min with varying concentrations of HU, similarly as described previously. [15] Subsequently, CDP was introduced in the reaction and incubated for 2 h to generate the necessary dCDP by the remaining active RNR Ia. After heat inactivation of RNR Ia, the mixture was supplied to a FLARE assay containing only dDTPs. Hence, the dCDP generated by RNR Ia during the pre‐incubation with HU determines the resulting fluorescent signal.

**Fig. 4:**
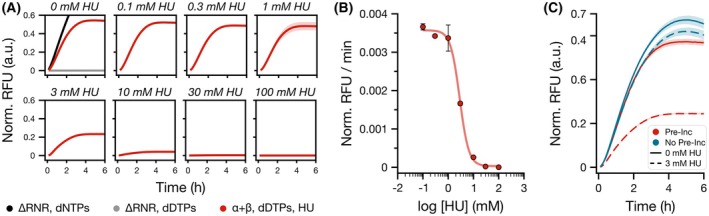
Quantification of hydroxyurea (HU) inhibition of RNR activity with FLARE. (A) Comparison of normalized relative fluorescence units (Norm. RFU) values over time of ribonucleotide reductase (RNR) Ia activity with 30‐min pre‐incubation with HU titration using a dCTP‐limiting set‐up (200 μm dDTPs added to the FLARE reactions). 10 μm of each RNR Ia subunit was pre‐incubated with increasing concentrations of HU (0–100 mm) and after heat inactivation, 1 μm of inhibited RNR Ia was added to the FLARE reactions. (B) Apparent rates of FLARE reactions (red circles) depending on different HU concentrations were fit to a 4‐parameter logistic dose–response model (red line). The determined apparent IC_50_ value for 10 μm RNR Ia and 30‐min pre‐incubation is ~2.8 mm. (C) Confirmation of the time and dose‐dependent response of RNR Ia to HU. Samples pretreated with HU are shown in red, while samples where HU was directly added to FLARE are shown in blue. Solid lines represent FLARE reactions with RNR Ia without HU treatment (0 mm HU), and dashed lines represent treatment with 3 mm HU. When RNR Ia is pretreated with 3 mm HU there is a clear reduction of the measured activity compared to the sample with 3 mm HU but without pretreatment. For all measurements: technical replicates; *n* = 3, mean and SD shown. Reaction measurements were normalized with min–max scaling between the averages of the maximum fluorescence readouts of ∆RNR‐dNTPs samples and t0 measurement of ∆RNR‐dDTPs‐CDP samples.

When RNR Ia was pretreated with HU, the maximum detected amplitude of fluorescence signals decreased gradually until no significant signal was observed at 30 mm HU (Fig. 4A). Similarly, the observed apparent rates decreased following a similar trend (Fig. S12) and could be used to determine the dose–response relationship between HU and RNR Ia with FLARE readout (Fig. 4B). Under these conditions, this allowed us to estimate an apparent IC_50_ value of ~2.8 mm HU after the 30‐min pre‐incubation of RNR Ia with HU and CDP substrate (i.e., IC_50_ ~ 280 μm for in‐assay conditions), which is in agreement with previously reported values for *E coli* RNR Ia. [52]

We further confirmed the time dependency of the inhibition of RNR Ia by HU by supplementing 3 mm of HU directly to a standard dCTP‐limiting FLARE set‐up and compared it to pre‐incubating the enzyme with HU (Fig. 4C). In this case, we observed a 55% reduction in apparent reaction rates when RNR Ia was pre‐incubated with 3 mm HU, compared to when RNR Ia was not pretreated with HU. In contrast, when supplying 3 mm HU directly to FLARE, we observed only a mild 8% reduction in reaction rates, which also indicated that the downstream enzymes in FLARE are not affected by higher HU concentrations. More notably, as a result of the slow kinetics of RNR Ia inactivation by HU, [52] in the samples without HU pre‐incubation, still active RNR Ia could generate sufficient dCTP for FLARE readout before inactivation. This effect is also partly due to the high sensitivity of the assay at low concentrations of active enzyme (Fig. S6) or low substrate concentrations (Fig. S7).

FLAREs real‐time properties could therefore be directly used to determine the dose response and time dependency of RNR Ia inhibitors with complex mechanisms of action. [52, 57] As such, FLARE might prove useful as an indirect readout to determine the dose‐dependent kinetics of RNR Ia's suicide inhibitors such as HU, or it might be directly compatible with identifying more powerful competitive inhibitors.

## Discussion

We developed FLARE (Fluorescent Light‐up Aptamer RNR Enzymatic assay), a novel assay for the detection in real‐time of RNR multiturnover activity based on the generation of specific dNTPs in reaction set‐ups depleted of those dNTPs. We designed FLARE as a rapid high‐throughput enzymatic assay that relies on widely available instrumentation and commercially available enzymes. FLARE is intended to complement traditional endpoint and kinetic quench‐flow assays and reduces the processing times compared to other available high‐throughput assays based on mass spectrometry [39] or PCR‐based [15] readouts. However, compared to other established assays based on kinetic quench‐flow measurements, FLARE does not allow for the direct determination of the activity of RNR Ia, but rather amplifies and reports the cumulative multiturnover activity by RNR Ia and allows the real‐time tracking of the coupled reactions required to elicit a fluorescent signal. The readout based on transcription of the Broccoli RNA FLAP depended on the orchestrated sequential catalytic activities of RNR Ia, NDK, Phi29 DNAP, and T7 RNAP. Fluorescent signal was generated only in the presence of both RNR Ia subunits, and it could be detected even at 10 nm of input RNR Ia. The increased sensitivity of the assay compared to other high‐throughput set‐ups enabled to detect multiturnover catalytic activity with multiple NDP substrates supplied simultaneously and to recreate the allosteric regulatory patterns reconstructed from *in vitro* mechanistic and structural studies.

While FLARE was developed using *E. coli* RNR class Ia as a model enzyme, we designed FLARE to be a modular system, which could be adapted to any class of RNRs with minor adjustments to the assay composition or by optimizing the assembly pathway required for other RNR classes. For instance, the reducing agent for regeneration of the catalytic site would have to be adjusted depending on the RNR class that is introduced in FLARE. While phosphines such as TCEP are compatible with several RNR classes, [47] they proved to be unable to regenerate the catalytic cysteines in class II RNR from *Stackebrandtia nassauensis*, which instead required optimizing the DTT concentration for *in vitro* activity. [58] Nonetheless, RNR‐dependent dNTP synthesis showed that disulfide reduction in the catalytic site is not the rate‐limiting step in FLARE primarily because we did not observe a major difference in the apparent rate of the FLARE reactions when the assay is performed with 100 nm or 1000 nm RNR Ia (Fig. S6), suggesting slower rates in the downstream reaction steps during DNA synthesis and/or transcription of the Broccoli FLAP.

When adapting the assay to screen for inhibition by hydroxyurea, RNR Ia pre‐incubated with HU exhibited a dose‐ and time‐dependent mode of inhibition by HU. This reflected the mechanism of action of HU, which relies on quenching of the Y_122_· in the presence of the α_2_β_2_ [56, 59]. Using FLARE's real‐time measurements, we confirmed HU's time‐dependent mechanism of inhibition, which allowed for a fraction of the enzyme population to complete one or several turnovers before the radical is destroyed, even at inhibitory concentrations of HU. The inhibition screening with HU, highlighted the high sensitivity of FLARE, which lends itself to screening time‐dependent inhibitory profiles of novel and potentially more potent inhibitory compounds of RNR Ia activity. Furthermore, FLARE has the potential to be used for screening of inhibitors with different inhibition mechanisms (e.g., competitive binding), rather than the formation of the radical cofactor. [2, 10] The coupled nature of FLARE would further enable to determine the effect of these compounds on DNA synthesis and transcription, by assessing the impact on the downstream polymerases involved in the assay.

The modular design of FLARE theoretically allows to integrate other readouts, using the wide array of available RNA fluorescent light‐up aptamers, and can be likely adapted for the activity of other known RNR classes. This therefore enables high‐throughput analyses of multiturnover catalytic activity of RNRs for both mechanistic studies and screening of inhibitors for novel therapies targeting RNR class Ia and other RNR classes.

## Author contributions

HM and JDC conceived the project and designed the experiments. JDC and NEN collected data. JDC performed relevant analyses and designed the figures. VG purified RNR subunits. All authors wrote the paper and approved the final manuscript for publication.

## Supporting information


**File S1.** Python script for the estimation of apparent rates of FLARE reactions.

**File S2.** Figure source data for results presented in this study.

**Fig. S1.** Titration of varying DFHBI‐1T concentrations in FLARE assay.
**Fig. S2.** Example image of estimation of FLARE apparent rate.
**Fig. S3.** Comparison of β2 radical formation using UV–vis dropline correction method at A_410_ nm.
**Fig. S4.** Characterization of RNR Ia activity with different concentration of TCEP as reducing agent using PCR‐based assay.
**Fig. S5.** Establishing proof‐of‐concept of FLARE assay using dCTP‐limiting set‐up.
**Fig. S6.** Titration of RNR Ia subunits in FLARE under dCTP‐limiting conditions.
**Fig. S7.** Titration of FLARE substrates in dCTP‐limiting set‐up.
**Fig. S8.** Confirmation of reduction of each individual NDP in the respective dNTP‐limiting set‐up.
**Fig. S9.** Comparison of FLARE signals depending on dUTP incorporation by Phi29 DNAP.
**Fig. S10.** Comparison of RNR Ia cascade ‘directions’ in FLARE conditions with different combinations of NDPs and dNTPs.
**Fig. S11.** Comparison of reduction of UDP and VDPs (i.e. ADP, GDP, CDP).
**Fig. S12.** Dose‐dependent inhibition of RNR Ia by hydroxyurea (HU) in FLARE, with pre‐incubation of RNR Ia with HU.
**Table S1.** Primers and ssDNA Oligos.
**Table S2.** Coding sequences of purified proteins.
**Table S3.** RNR reaction buffer composition.
**Table S4.** rNTP Mix.
**Table S5.** Broccoli‐based assay reaction composition. Example composition with CDP and dDTP Mix. Concentrations also apply to other NDPs and dNTPs mixes.

## Data Availability

The data that support the findings of this study are available in the supplementary material of this article. The source data for the data presented in this study can be found in File S2.
