# Supplementary material for: Real‐time assay of ribonucleotide reductase activity with a fluorescent RNA aptamer
Source: FEBS Lett. 2025 Dec 1;600(4):467–80. doi: 10.1002/1873-3468.70237 (PMC12926857; doi:10.1002/1873-3468.70237)
Supplement: Supplementary file 3 — Fig. S1. Titration of varying DFHBI‐1T concentrations in FLARE assay. Fig. S2. Example image of estimation of FLARE apparent rate. Fig. S3. Comparison of β2 radical formation using UV–vis dropline correction method at A410 nm. Fig. S4. Characterization of RNR Ia activity with different concentration of TCEP as reducing agent using PCR‐based assay. Fig. S5. Establishing proof‐of‐concept of FLARE assay using dCTP‐limiting set‐up. Fig. S6. Titration of RNR Ia subunits in FLARE under dCTP‐limiting conditions. Fig. S7. Titration of FLARE substrates in dCTP‐limiting set‐up. Fig. S8. Confirmation of reduction of each individual NDP in the respective dNTP‐limiting set‐up. Fig. S9. Comparison of FLARE signals depending on dUTP incorporation by Phi29 DNAP. Fig. S10. Comparison of RNR Ia cascade ‘directions’ in FLARE conditions with different combinations of NDPs and dNTPs. Fig. S11. Comparison of reduction of UDP and VDPs (i.e. ADP, GDP, CDP). Fig. S12. Dose‐dependent inhibition of RNR Ia by hydroxyurea (HU) in FLARE, with pre‐incubation of RNR Ia with HU. Table S1. Primers and ssDNA Oligos. Table S2. Coding sequences of purified proteins. Table S3. RNR reaction buffer composition. Table S4. rNTP Mix. Table S5. Broccoli‐based assay reaction composition. Example composition with CDP and dDTP Mix. Concentrations also apply to other NDPs and dNTPs mixes. [file FEB2-600-467-s001.pdf]

Real-time assay of ribonucleotide reductase activity with a fluorescent RNA aptamer

Jacopo De Capitani, [1] Noemi E. Nwosu, [1] Viktoria Gocke, [2] Müge Kasanmascheff, * [2] Hannes Mutschler * [1]

1. Biomimetic Chemistry, Department of Chemistry and Chemical Biology, TU Dortmund University, Dortmund, 44227, Germany
2. Physical Chemistry, Department of Chemistry and Chemical Biology, TU Dortmund University, Dortmund, 44227, Germany

*** Corresponding author:** hannes.mutschler@tu-dortmund.de

Supporting Information

Supplementary Tables

DNA constructs and sequences

Table S1: Primers and ssDNA Oligos.

Primer Number / Oligo Name	Sequence
pr01 (NDK isolation FWD)	TTCACCTTAACTTTAAGAAGGAGATATACCATGAGAGGATCGCAT<br>CACCAT
pr02 (NDK isolation REV)	ACAGCCAAGCTTCGTAAATTCTATACAAAATTAACGGGTGCGCGG<br>GCACAC
pr03 (pBAD33 backbone FWD)	TTTTGTATAGAATTTACGAAGCTTGGCTGT
pr04 (pBAD backbone REV)	GGATCCGTGATGGTGATGGTGATGCGATCCTCTCAT
Broccoli ssDNA	GAAATTAATACGACTCACTATAGGGAGACGGTCGGGTCCAGATAT<br>TCGTATCTGTCGAGTAGAGTGTGGGCTCCC
pr05 (REV Broccoli)	GGGAGCCCACACTCTACT
pr06 (pUC19 PCR Assay FWD)	CCCAGTCACGACGTTGTAAAACG
pr07 (pUC19 PCR Assay REV)	AGCGGATAACAATTTCACACAGG

Table S2: Coding sequences of purified proteins.

Coding Sequence	Sequence
Nucleoside Diphosphate Kinase (NDK)	atgagaggatcgcatcaccatcaccatcacggatccgatgacgatgacaaagctattgaacgtactttttccatcatcaa<br>accgaacgcggtagcaaaaaacgtcattggtaatatctttgcgcgctttgaagctgcagggttcaaaattgttggcacca<br>aaatgctgcacctgaccgttgaacaggcacgtggcttttatgctgaacacgatggaaaaccgttctttgatggtctggtt<br>gaattcatgacctctggcccgatcgtggtttccgtgctggaaggtgaaaacgccgttcagcgtcaccgcgatctgctggg<br>cgcgaccaatccggcaaacgcactggctggtactctgcgcgctgattacgctgacagcctgaccgaaaacggtacccacg<br>gttctgattccgtcgaatctgccgctcgcgaaatcgcttatttctttggcgaaggcgaagtgtgcccgcgcacccgttaa
RNR 1a Alpha (His6) (*nrdA*)	atgagaggatcgcatcaccatcaccatcacggatccaatcagaatctgctggtgacaaagcgcgacggtagcacagagcg<br>catcaatctcgacaaaatccatcgcgttctggattgggcggcagaaggactgcataacgtttcgatttcccaggtcgagc<br>tgcgctcccacattcagttttatgacggtatcaagacctctgacatccacgaaaccattatcaaggctgccgcagacctg<br>atctcccgtgatgcgccggattatcagtatctcgccgcgcgcctggcgatcttccacctgcgtaaaaaagcctacggcca<br>gtttgagccgcctgcgctgtacgaccacgtggtgaaaatggtcgagatgggcaaatacgataatcatctgctggaagact<br>acacggaagaagagttcaagcagatggacacctttatcgatcacgaccgtgatatgaccttctcttatgctgccgttaag<br>cagctggaaggcaaatatctggtacagaaccgcgtgaccggcgaaatctatgagagcgcccagttcctttatattctagt<br>tgccgcgtgcttgttctcgaactacccgcgtgaaacgcgcctgcaatatgtgaagcgtttttacgacgcggtttccacat<br>ttaaaatttcgctgccgacgccaatcatgtccggcgtgcgtaccccgactcgtcagttcagctcctgcgtactgatcgag<br>tgcggtgacagcctggattccatcaacgccacctccagcgcgattgttaaatacgtttcccagcgtgccgggatcggcat<br>caacgccgggcgtattcgtgcgctgggtagcccgattcgcggtggtgaagcgttccataccggctgcattccgttctaca<br>aacatttccagacagcggtgaaatcctgctctcagggcggtgtgcgcggcggtgcggcaacgctgttctacccgatgtgg<br>catctggaagtggaaagcctgctggtgttgaaaaacaaccgtggtgtggaaggcaaccgcgtgcgtcatatggactacgg<br>ggtacaaatcaacaaactgatgtatacccgtctgctgaaaggtgaagatatcaccctgttcagcccgtccgacgtaccgg<br>ggctgtacgacgcgttcttcgccgatcaggaagagtttgaacgtctgtataccaaatatgagaaagacgacagcatccgc<br>aagcagcgtgtgaaagccgttgagctgttctcgctgatgatgcaggaacgtgcgtctaccggtcgtatctatattcagaa<br>cgttgaccactgcaatacccatagcccgtttgatccggccatcgcgccagtgcgtcagtctaacctgtgcctggagatag<br>ccctgccgaccaaaccgctgaacgacgtcaacgacgagaacggtgaaatcgcgctgtgtacgctgtctgctttcaacctg<br>ggcgcaattaataacctggatgaactggaagagctggcaattctggcggttcgtgcacttgacgcgctgctggattatca<br>ggattacccgatcccggccgccaaacgtggagcgatgggtcgtcgtacgctgggtattggtgtgatcaacttcgcttact<br>acctggcgaagcacggtaaacgctactccgacggcagcgccaacaacctgacgcataaaaccttcgaagccattcagtat<br>tacctgctgaaagcctctaatgagctggcgaaagagcaaggcgcgtgcccgtggtttaacgaaaccacttacgcgaaagg

	gatcctgccgatcgatacctataagaaagatctggataccatcgctaatgagccgctgcattacgactgggaagctctgc<br>gtgagtcaatcaaaacgcacggtctgcgtaactccacgctttctgctctgatgccgtccgagacttcttcgcagatctct<br>aacgccactaacggtattgaaccgccgcgcggttacgtcagcatcaaagcgtcgaaagacggtattttgcgccaggtggt<br>gccggactacgagcacctgcacgacgcctatgagctgctgtgggaaatgccgggtaacgatggttatctgcaactggtgg<br>gtatcatgcagaaatttatcgatcagtcgatctctgccaacaccaactacgatccgtcacgcttcccgtcaggaaaagtg<br>ccgatgcagcagttgctgaaagacctgctcaccgcctacaaattcggggtcaaaacactgtattatcagaacacccgtga<br>cggcgctgaagacgcacaagacgatctggtgccgtcaatccaggacgatggctgcgaaagcggcgcatgtaagatctga
RNR 1a Beta (*nrdB*)	atggcatataccaccttttcacagacgaaaaatgatcagctcaaagaaccgatgttctttggtcagccggtcaacgtggc<br>tcgctacgatcagcaaaaatatgacatcttcgaaaagctgatcgaaaagcagctctctttcttctggcgtccggaagaag<br>ttgacgtctcccgcgaccgtatagattaccaggcgctgccggagcacgaaaaacacatctttatcagcaacctgaaatat<br>cagacgctgctggattccattcagggtcgtagcccgaacgtggcgctattgccgcttatttctattccggaactggaaac<br>ctgggtcgaaacctgggcgttctcagaaacgattcattcccgttcctatactcatatcattcgtaatatcgttaacgatc<br>cgtctgttgtgtttgacgatatcgtcaccaacgagcagatccagaaacgtgcggaagggatctccagctattacgatgag<br>ctgatcgaaatgaccagctactggcatctgctgggcgaaggtacccacaccgttaacggtaaaactgtgaccgttagcct<br>gcgcgagctgaagaaaaaactgtatctctgcctgatgagcgttaacgcgctggaagcgattcgtttctacgtcagctttg<br>cttgttccttcgcatttgcagaacgcgaattgatggaaggcaacgccaaaattattcgcctgattgcccgcgacgaagcc<br>ctgcacctgaccggcacccagcatatgctgaatctgctgcgcagcggcgcggacgatcctgagatggcggaaattgccga<br>agagtgtaagcaggagtgctatgacctgtttgttcaggcagctcaacaggagaaagactgggcggattatctgttccgcg<br>acggttcgatgattggtctgaataaagacattctctgccagtacgttgaatacatcaccaatatccgtatgcaggcagtc<br>ggtttggatctgccgttccagacgcgctccaacccgatcccgtggatcaacacttggctggtgtctgataacgtgcaggt<br>tgctccgcaggaagtggaagtcagttcttatctggtcgggcagattgactcggaagtggacaccgacgatttgagtaact<br>tccagctctga

Composition of FLARE assay

Table S3: RNR reaction buffer composition.

Component	1x Concentration
Tris-HCl pH 7.5	50 mM
$Mg(OAc)_2$	30 mM
DTT	1.5 mM
NaCl	10 mM
Spermidine	2 mM

Table S4: rNTP Mix

Component	7.5x Concentration
ATP	37.5 mM
GTP	15 mM
CTP	15 mM
UTP	15 mM

Table S5: Broccoli-based assay reaction composition. Example composition with CDP and dDTP Mix. Concentrations also apply to other NDPs and dNTPs mixes.

Component	Stock Concentration	Final Concentration
10x RNR Reaction Buffer	10x	1x
ssDNA Broccoli, pr05 (equimolar mix)	10 µM	0.5 µM
rNTP Mix	7.5x	1x
TCEP	200 mM	1.5 mM
CDP	200 mM	2 mM
dDTPs (equimolar mix dA, dG, dT)	10 mM	0.2 mM
Murine Rnase Inhibitor (NEB)	40 U/µL	1 U/µL
NDK	5.77 µM	0.115 µM
T7 RNA Polymerase (ThermoFisher)	7.48 µM	0.2 µM
NrdA	30 µM	1 µM
NrdB	20 µM	1 µM
Phi29 DNA Polymerase	10 U/µL	0.67 U/µL
DFHBI-1T	1.5 mM	0.01 mM

Supplementary Figures

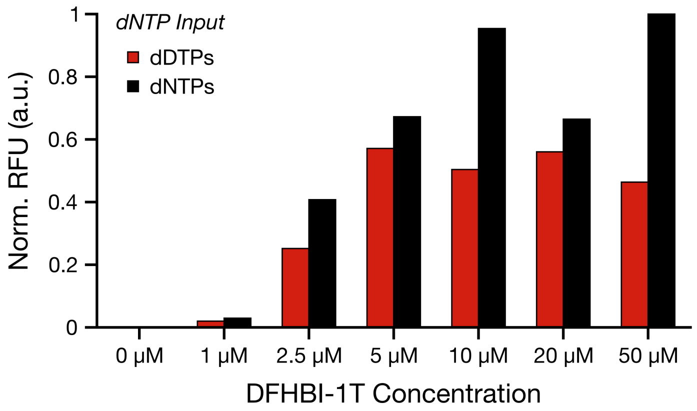

Figure S1: Titration of varying DFHBI-1T concentrations in FLARE assay.

The FLARE assay was used in a dCTP-limiting set-up (2 mM CDP with 200 µM dDTPs) to screen different concentrations (0-50 µM) of DFHBI-1T fluorophore and establish the optimal DFHBI-1T concentration. Maximum amplitude of each reaction shown here after incubation of 6 hours. RFU values normalized with min-max scaling between maximum and minimum measured values.

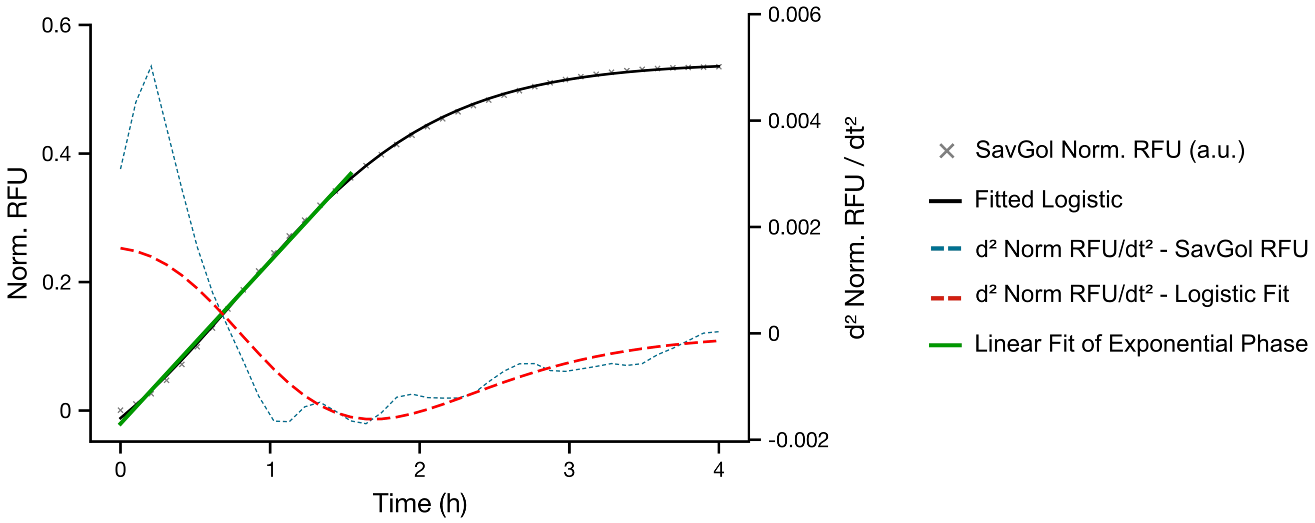

Figure S2: Example image of estimation of FLARE apparent rate.

Normalized RFU data is smoothened with Savitzky–Golay filter (gray crosses). The smoothened data is fit to a scaled logistic function (black) and the second derivative is computed (red - here compared with central difference of smoothened data in blue). The local maximum and minimum of the second derivative are used to define x coordinates between which to fit a linear regression model of the smoothened data (green). The apparent rate of the reaction is defined as the slope of the linear model and represents the rate of increase of fluorescent signal generated as part of the coupled assay. It therefore is representative of the combined activity of RNR Ia, NDK, Phi29 DNAP and T7 RNAP, but is not a direct representation of the activity of RNR Ia.

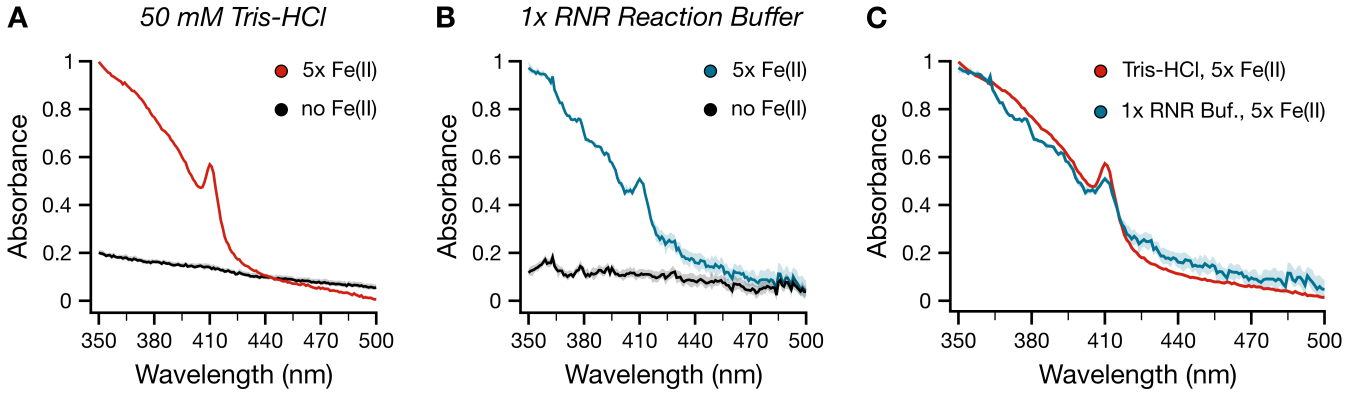

Figure S3: Comparison of β2 radical formation using UV-vis dropline correction method at A_{410} nm.

(A) Formation of β2 Y_{122}• radical in 50 mM Tris-HCl pH 7.5, used as reference for optimal conditions, with either no iron(II) (black) or 5 molar equivalents of iron(II) (red). Based on the dropline correction method, 61% of β2 showed formation of Y_{122}• radical. (B) Formation of β2 Y_{122}• radical in 1x RNR Reaction Buffer with either no iron(II) (black) or 5 molar equivalents of iron(II) (blue). 49% of β2 showed formation of Y_{122}• radical. (C) Comparison spectra of samples with 5 equivalents of iron(II). The observed values of Fe^{2+} loading in β2 are consistent with previously reported values, which show that β2 activity is limited to at most 60% active β2 and with at most 3.6 eq of iron atoms bound, regardless of Fe^{2+} availability. [44, 45] Using this established technique, we could verify the correct loading of the Fe^{2+} metallocofactor in the β2 dimer in the buffer composition required for the FLARE assay. For all measurements, technical replicates, n=3, mean and s.d. are shown. Some standard deviations in the graphs may not be visible due to small magnitude.

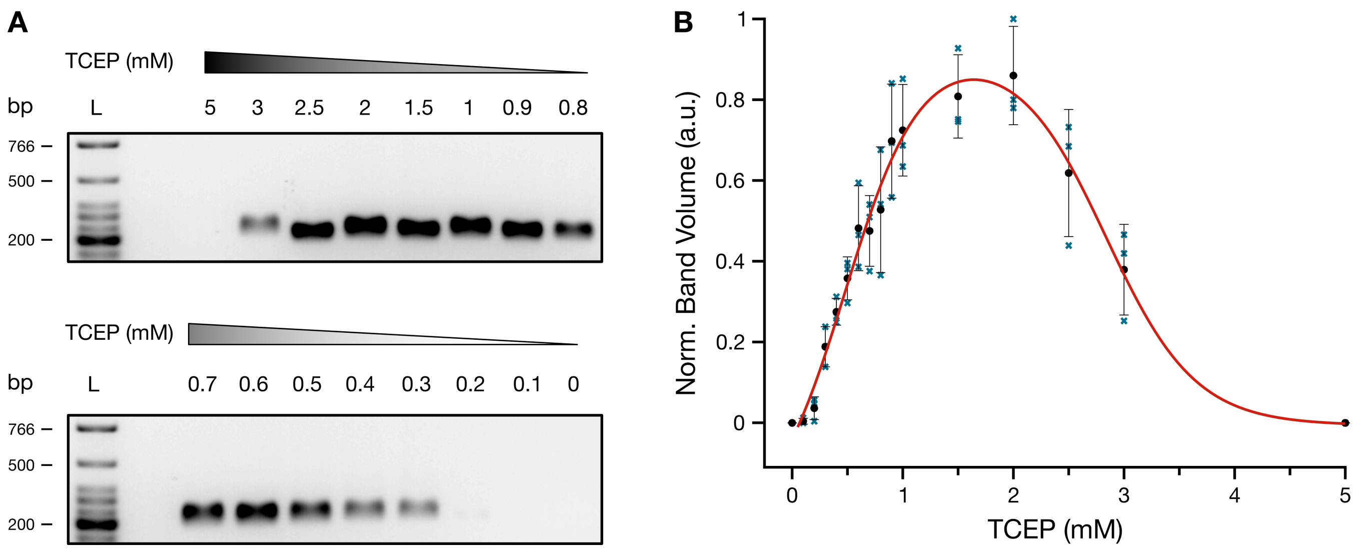

Figure S4: Characterization of RNR Ia activity with different concentration of TCEP as reducing agent using PCR-based assay.

A PCR-based assay [15] was used in a dCTP-limiting set-up (2 mM CDP with 200 µM dDTPs) to determine the optimal concentration for multi-turnover activity of RNR Ia in a coupled assay. (A) Example 3% agarose gel for imaging of PCR-based assay reactions. Gel was strained with SYBR Safe (Invitrogen) and Low Molecular Weight Ladder (NEB) was used as marker. Expected PCR product length 137 bp. (B) Normalized band volume from measurements of PCR-based assay. Independent replicates fit to biphasic dose-response model (Eq 2) In this coupled assay, RNR Ia exhibited an activity profile that depended on the TCEP concentration. This confirmed that 1.5 mM TCEP, alongside 1.5 mM DTT, could act as reducing agents for the regeneration of the catalytic cysteines in the α subunit. [47] For all measurements, independent replicates, n=3, mean and s.d. are shown. Band volumes were normalized with min-max scaling between the maximum and minimum band intensities.

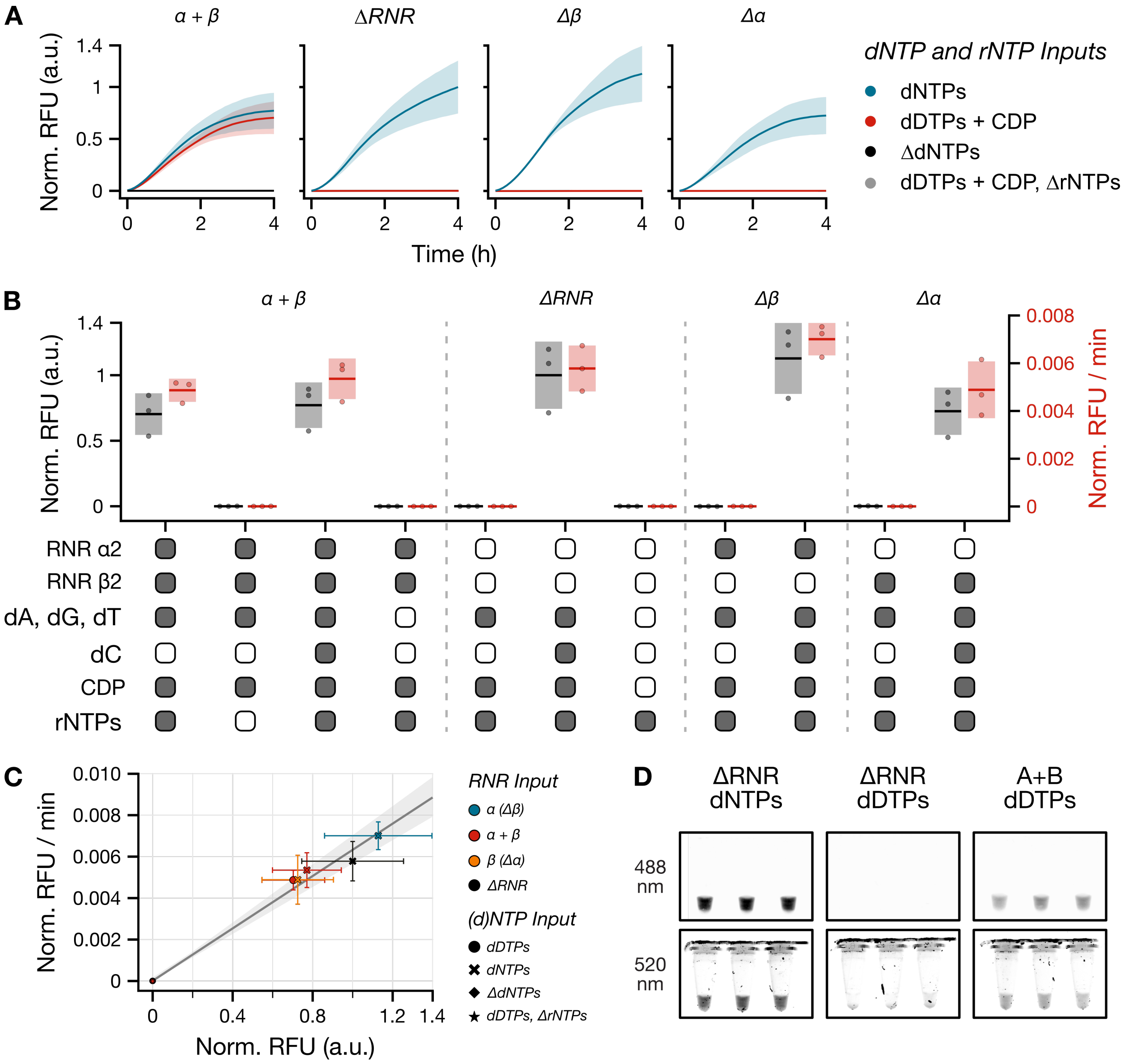

Figure S5: Establishing proof-of-concept of FLARE assay using dCTP-limiting set-up.

RFU measurements of proof-of-concept testing with full control set using a dCTP-limiting set-up (2 mM CDP along with 200 µM dDTPs). When RNR Ia subunits were included in the reactions, 1 µM of each subunit was included. (A) Real-time normalized RFU values. (B) Apparent rates (red) of reactions, compared with maximum normalized RFU amplitude (black). (C) Normalized maximum RFU amplitude correlates with increasing apparent rate (Pearson R = 0.99), indicating that faster reaction kinetics lead to higher RFU signals. Point plot shows mean and s.d. of both variables; linear regression shows 95% confidence interval of the heteroscedastic least-squares fit. (D) Example reaction tubes for qualitative assessment of reactions, imaged in Azure Biosystems Sapphire with excitation at 488 nm and at 520 nm. FLARE could be used to detect and amplify the activity of RNR Ia only in the presence of both subunits. For all panels, independent replicates, n=3, mean and s.d. are shown. For panels, A, B and C, reaction measurements were normalized with min-max scaling between the averages of the maximum fluorescence read-outs of ΔRNR-dNTPs samples and t0 measurement of ΔRNR-dDTPs-CDP samples.

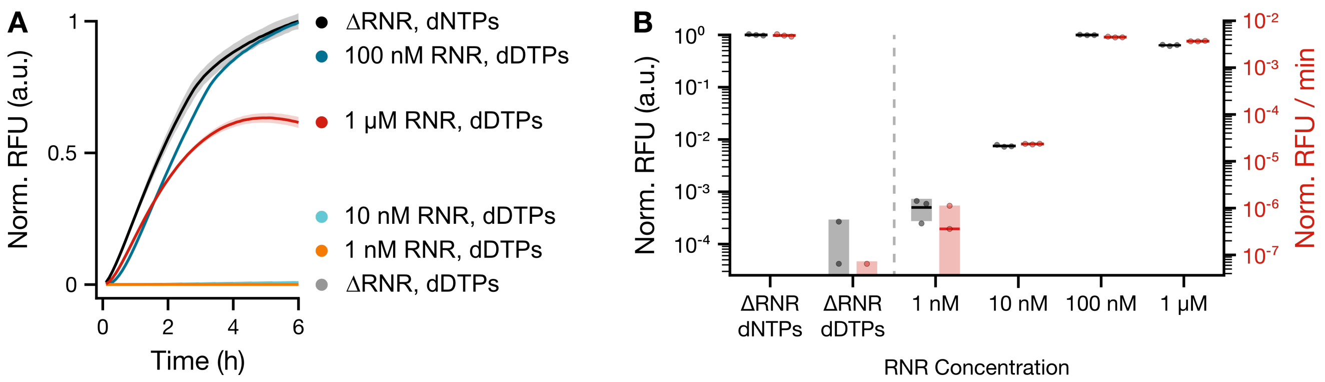

Figure S6: Titration of RNR Ia subunits in FLARE under dCTP-limiting conditions.

RFU measurements of linear 10-fold titration of RNR 1a (1 µM - 1 nM) to determine the detection range of FLARE based on available RNR Ia. Reactions were carried out for 6 hours under dCTP-limiting conditions (2 mM CDP along with 200 µM dDTPs). (A) Real-time normalized RFU values. (B) Apparent rates (red) of reactions with titration of RNR 1a, compared with maximum normalized RFU amplitude (black). By gradually reducing the concentration of RNR Ia, we observed that FLARE could be used to amplify the activity of RNR Ia down to 10 nM of each RNR Ia subunits. For all measurements, technical replicates, n=3, mean and s.d. are shown. Reaction measurements were normalized with min-max scaling between the averages of the maximum fluorescence read-outs of ΔRNR-dNTPs samples and t0 measurement of ΔRNR-dDTPs-CDP samples.

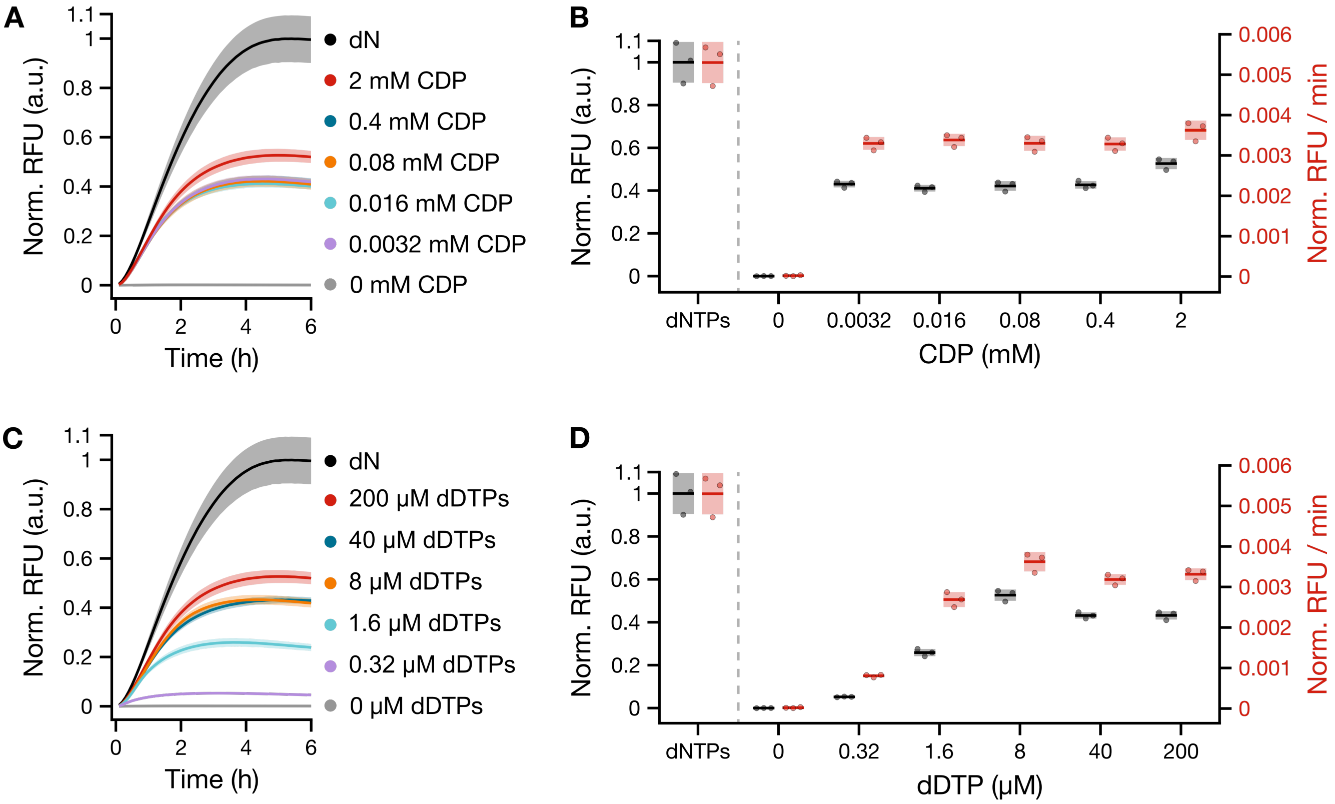

Figure S7: Titration of FLARE substrates in dCTP-limiting set-up.

A dCTP-limiting set-up of FLARE was challenged with decreasing concentrations of either CDP or dDTPs to determine the detection range of the assay under different substrate concentrations. When CDP was titrated (2 mM – 3.2 μM), 200 μM dDTPs were consistently supplied. When dDTPs were titrated (200 μM – 0.32 μM), 2 mM CDP were consistently supplied. (A) Real-time normalized RFU values of FLARE detection with decreasing CDP concentrations. (B) Apparent rates (red) of reactions with titration of CDP, compared with maximum normalized RFU amplitude (black). (C) Real-time normalized RFU values of FLARE detection with decreasing dDTPs concentrations. (D) Apparent rates (red) of reactions with titration of dDTPs, compared with maximum normalized RFU amplitude (black). By titrating the concentrations of available FLARE substrates, we observed that FLARE is extremely sensitive to low concentrations of available RNR Ia substrate (i.e. CDP) and can detect and amplify RNR Ia activity even at low CDP concentrations. Similarly, FLARE showed high sensitivity to low concentrations of available dDTPs, showing consistent apparent rates down to 8 μM and unambiguous detection down to 0.32 μM dDTPs. For all measurements, technical replicates, n=3, mean and s.d. are shown. Reaction measurements were normalized with min-max scaling between the averages of the maximum fluorescence read-outs of ΔRNR-dNTPs samples and t0 measurement of ΔRNR-dDTPs-CDP samples.

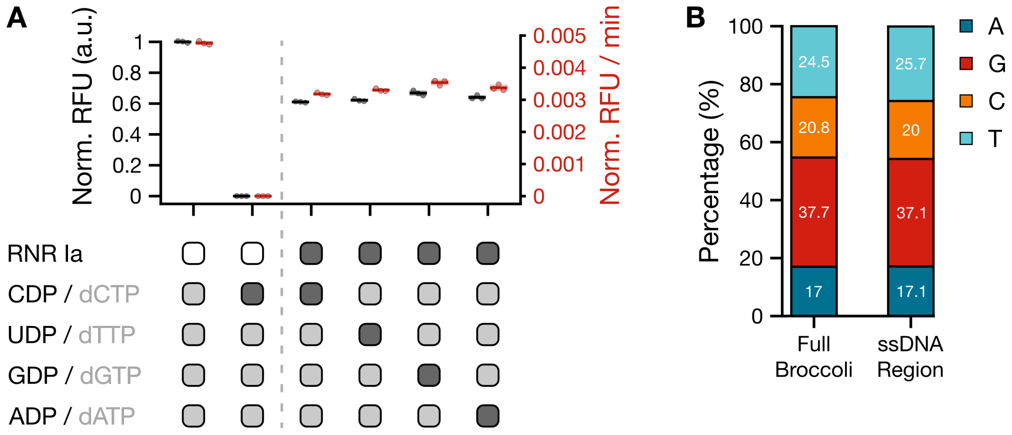

Figure S8: Confirmation of reduction of each individual NDP in the respective dNTP-limiting set-up. The FLARE assay was used in different dNTP-limiting set-ups to test whether if it could be used to detect and amplify the reduction by RNR Ia of each individual NDP. Reactions where therefore run with individual NDPs in the respective dNTP-limiting set-ups (e.g. 2 mM CDP with 200 µM dDTPs; or 2 mM ADP with 200 µM dBTPs; etc.) (A) Maximum normalized RFU measurements (black) and respective apparent reaction rates (red) for different individual NDPs and remaining dNTPs supplied to assay reaction. In the sample matrix, NDPs supplied to the reactions are marked in black, while dNTPs in gray. We observed that the FLARE assay could be used to detect the RNR-dependent reduction of each NDP individually and that the assay detection was agnostic to which NDP had to be reduced for the fluorescent signal to be elicited. For panel A, technical replicates, n=3, mean and s.d. are shown. Reaction measurements were normalized with min-max scaling between the averages of the maximum fluorescence read-outs of ΔRNR-dNTPs samples and t0 measurement of ΔRNR-dDTPs-CDP samples). (B) Nucleotide composition of both the full-length Broccoli FLAP DNA template and the ssDNA region that is extended by Phi29 DNAP.

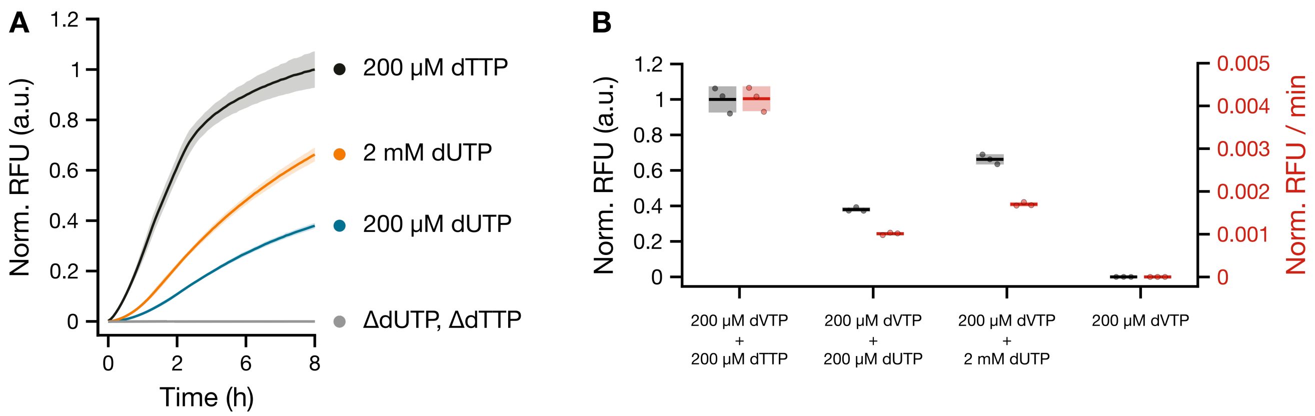

Figure S9: Comparison of FLARE signals depending on dUTP incorporation by Phi29 DNAP.
FLARE reactions were carried out in an RNR-independent fashion, therefore dUTP incorporation rate by Phi29 DNAP RFU signals in all samples were independent of RNR Ia activity (i.e. ΔRNR). All samples were supplied with 200 µM dVTP (dATP, dGTP, dCTP) and supplied with either 200 µM dTTP, 200 µM dUTP or 2 mM dUTP. RFU signals were measured for 8 hours. (A) Real-time normalized RFU values of dVTP samples supplied with either 200 µM dTTP (black), 200 µM dUTP (blue), 2 mM dUTP (orange) or no dUTP or dTTP (i.e. dVTPs only – gray). (B) Apparent rates (red) compared with maximum normalized RFU amplitude (black). We observed that Phi29 DNAP requires higher concentrations of available dUTP to generate a signal comparable to other dNTP-limiting assay conditions in FLARE. For all measurements, technical replicates, n=3, mean and s.d. are shown. Reaction measurements were normalized with min-max scaling between the averages of the maximum fluorescence read-outs of ΔRNR-dVTPs-dTTP samples and t0 measurement of ΔRNR-dVTPs samples.

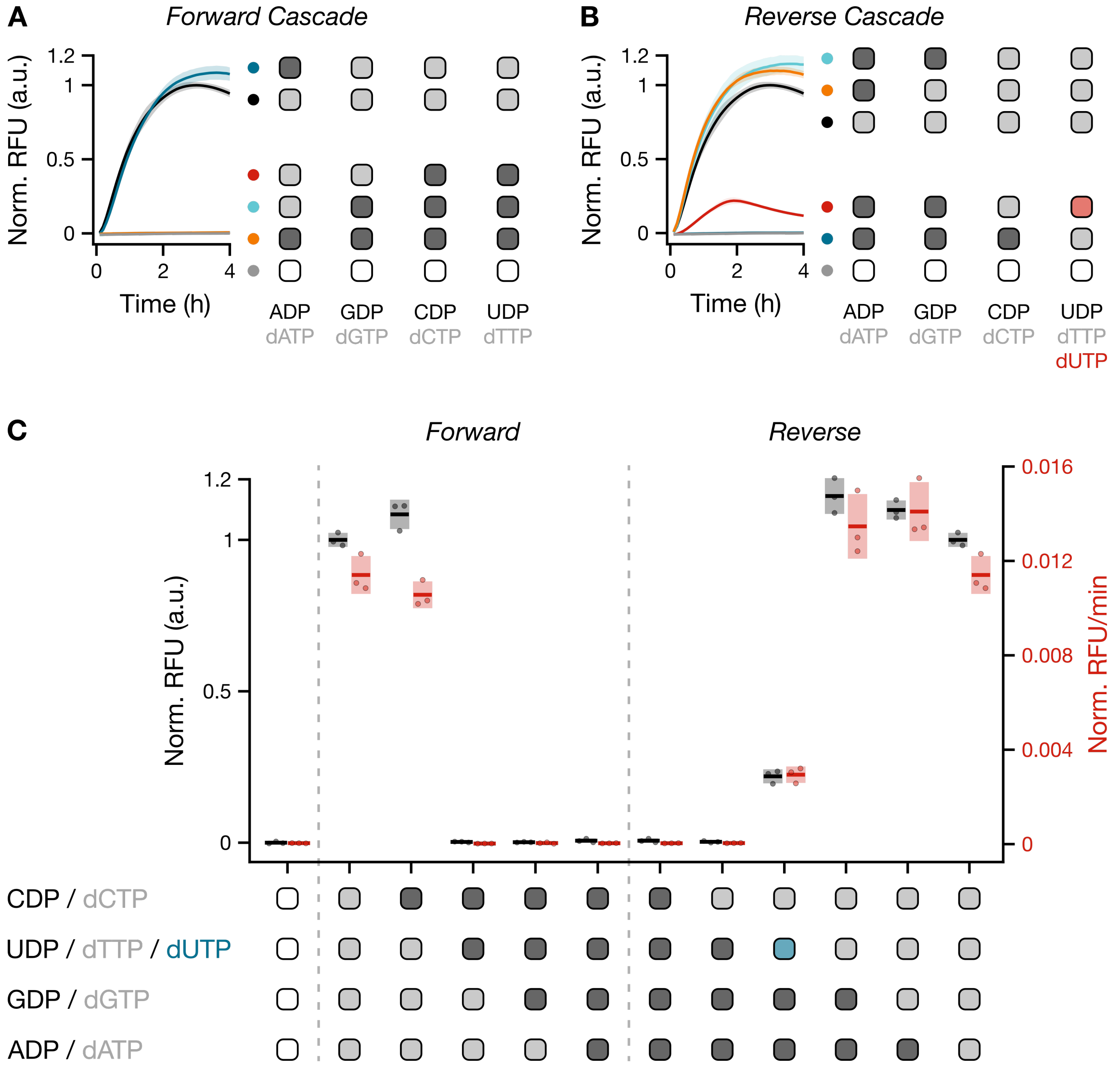

Figure S10: Comparison of RNR Ia cascade "directions" in FLARE conditions with different combinations of NDPs and dNTPs.

RFU measurements of simulated RNR reduction cascade of different NDPs, obtained by sequentially modifying the composition of the NDP and dNTPs supplied to the FLARE reactions. 2 mM of NDPs are supplied alongside 200 µM of dNTPs (e.g. when CDP and UDP must be reduced, 2 mM CDP and 2 mM UDP are supplied, alongside 200 µM dGTP and 200 µM dATP). (A) Real-time normalized RFU values of forward cascade and of (B) reverse cascade. (C) Apparent rates (red) of reactions simulating RNR reduction *forward* and *reverse* cascades, compared with maximum normalized RFU amplitude (black). In the sample matrices, NDPs supplied to the reactions are marked in black, while dNTPs in gray. When 200 µM dUTP are supplied instead of 200 mM dTTP, the box is marked in blue. FLARE reactions could therefore be used to replicate the allosteric regulatory pattern observed in mechanistic studies, [13] where dTTP acts as the specificity effector for the reduction of GDP. The specificity allosteric site seems to show a lower affinity for available dUTP, which could be used with lower affinity as effector for GDP reduction. For all measurements, independent replicates, n=3, mean and s.d. are shown. Reaction measurements were normalized with min-max scaling between the averages of the maximum fluorescence read-outs of dNTPs samples and t0 measurement of ΔdNTPs samples.

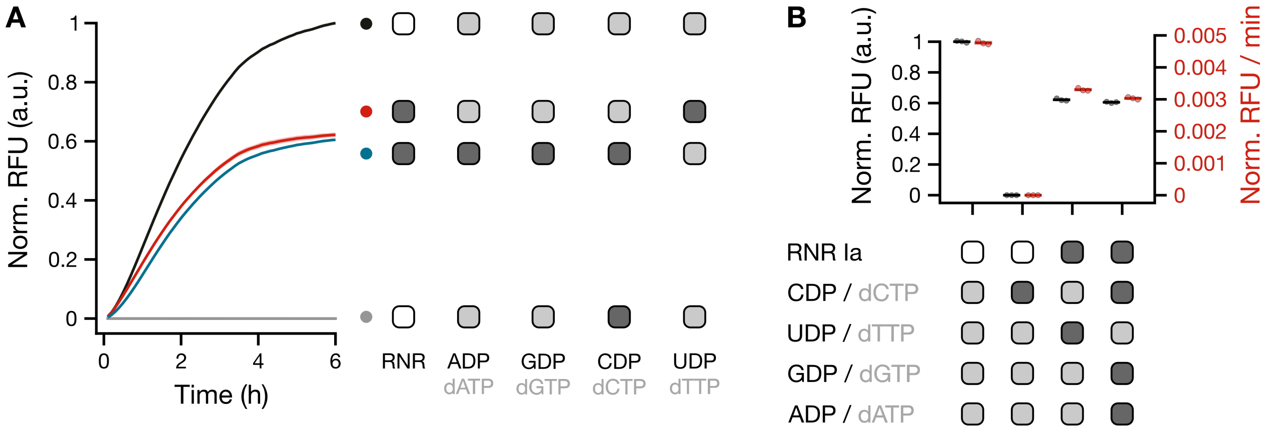

Figure S11: Comparison of reduction of UDP and VDPs (i.e. ADP, GDP, CDP).

To establish whether we could detect the RNR-dependent reduction of 3 NDPs, circumventing the limitations of the allosteric regulation of RNR Ia, we compared the reduction of UDP alone (i.e. reactions with 2 mM UDP and 200 µM dVTPs) with the reduction of VDPs (i.e. reactions with 2 mM of each ADP, GDP, CDP, along with 200 µM dTTP). (A) Real-time normalized RFU values. (B) Maximum normalized RFU measurements (black) and respective apparent reaction rates (red) for different individual NDPs and remaining dNTPs supplied to assay reaction. In the sample matrices, NDPs supplied to the reactions are marked in black, while dNTPs in gray. We observed that FLARE could be used to amplify the RNR-dependent reduction activity of all 3 VDPs, which was comparable to the reduction of UDP alone. This further confirmed that FLARE could be used to confirm the allosteric regulatory mechanism of RNR Ia for the use of dTTP as specificity effector for GDP reduction, instead of dUTP. For all measurements, technical replicates, n=3, mean and s.d. are shown. Reaction measurements were normalized with min-max scaling between the averages of the maximum fluorescence read-outs of ΔRNR-dNTPs samples and t0 measurement of ΔRNR-dDTPs-CDP samples.

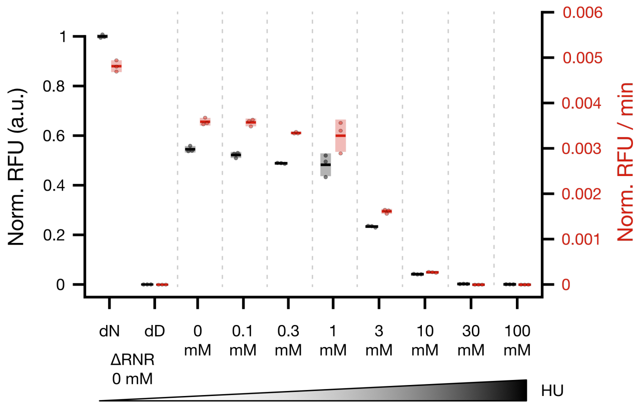

Figure S12: Dose-dependent inhibition of RNR Ia by hydroxyurea (HU) in FLARE, with pre-incubation of RNR Ia with HU.

FLARE was used to determine if it could be adapted for detection of dose-response of inhibition of RNR Ia by radical quenching by hydroxyurea (HU) RFU measurements of linear 10-fold titration of hydroxyurea (HU) (0 - 100 mM) were incubated with 10 µM of both subunits of RNR 1a for 30 min prior to real-time assay. After pre-incubation, 1 µM of treated RNR Ia was introduced to FLARE assay reactions, which were carried out for 6 hours. Maximum amplitude of RFU values shown in black and reaction apparent rate shown in red. FLARE could be adapted to determine whether RNR Ia was inhibited by HU. We observed a dose-dependent inhibition when RNR Ia was pre-incubated with HU for 30 min, which suggested that HU acts on a time and concentration-dependent fashion as a radical scavenger for RNR Ia. For all measurements, technical replicates, n=3, mean and s.d. are shown. Reaction measurements were normalized with min-max scaling between the averages of the maximum fluorescence read-outs of ΔRNR-dNTPs samples and t0 measurement of ΔRNR-dDTPs-CDP samples.
